# Extension and validation of a physiologically based toxicokinetic model for risk assessment of aluminium exposure in humans

**DOI:** 10.1007/s00204-025-04031-1

**Published:** 2025-04-19

**Authors:** Niklas Hartung, Gaby Wangorsch, Wilhelm Huisinga, Karin Weisser

**Affiliations:** 1 Institute of Mathematics, University of Potsdam, Potsdam, Germany; 2 Paul-Ehrlich-Institut (Federal Institute for Vaccines and Biomedicines), Langen, Germany

**Keywords:** PBTK, Toxicokinetics, Aluminium, Validation

## Abstract

The safety of aluminium (Al) exposure from sources such as food, parenteral nutrition or adjuvanted medicinal products is still a matter of uncertainty. Since toxicokinetic studies in humans are lacking, model predictions are warranted for risk assessment. Recently, we established a physiologically based toxicokinetic (PBTK) model for Al built on a comprehensive toxicokinetic $^{26}\textrm{Al}$ database, which could describe Al biokinetics in rats and human adults after single oral and intravenous doses of soluble Al salts. Since then, we have substantially amended the model, rendering it applicable to accurately represent children and their dynamically changing physiology (including maturating renal function in neonates and increased bone turnover during puberty). Also, additional sources of exposure were implemented, including vaccinations, subcutaneous allergen immunotherapies, food, antacids and parenteral nutrition. The model predictions in plasma and tissues were then compared to own published data and literature Al measurements after exposure from food (human reference values), parenteral nutrition (toxic levels in children and adults), adjuvanted allergen products or vaccines in rats and humans, and whole-body retention data. Al levels were predicted remarkably well, in plasma and toxicologically important tissues like bone, liver and brain. To our knowledge, this is the first Al PBTK model in humans ready for use in regulatory risk assessment, allowing to simulate Al exposure in children and adults from various sources of Al exposure like food and drinking water, Al contaminations in parenteral nutrition solutions, or poorly soluble Al complexes in medicinal products including Al-adjuvanted immunotherapeutics and vaccines.

**Supplementary Information:**

The online version contains supplementary material available at 10.1007/s00204-025-04031-1.

## Introduction

Aluminium (Al) is contained in adjuvants used in many vaccine and allergen immunotherapy products to enhance immune responses (Lindblad 2004). Furthermore, Al salts are used as active substance in antacids (Washington and Wilson 1986), but can also be present as contaminants in parenteral nutrition (PN) solutions (Kruger et al. 2014). At elevated levels of exposure, Al has toxic effects on bone, leading to a softening of the bones called osteomalacia, as well as on liver and the central nervous system (Krewski et al. 2007). For these reasons, a detailed understanding of Al toxicokinetics (TK) is crucial to evaluate the risk profile of Al-containing products.

Physiologically based toxicokinetic (PBTK) modelling allows to mechanistically translate external exposure to toxins into internal exposure in different tissues, thereby providing a quantitative framework for risk assessment (Dixit et al. 2003). We recently developed a PBTK model for Al, built on a comprehensive database of $^{26}\textrm{Al}$ tracer studies (Hethey et al. 2021). In contrast to the naturally occurring isotope $^{27}\textrm{Al}$, the radioisotope $^{26}\textrm{Al}$ can be quantified without background and in low amounts, which allowed for a parametrization of absorption and tissue uptake/release processes. This model was able to describe Al biokinetics in rats and human adults after single oral (p.o.) and intravenous (i.v.) doses of soluble Al salts like Al citrate or chloride. It was, however, not yet applicable for simulation of Al exposure from Al-containing products in the following aspects: (i) accurate representation of children and their dynamically changing physiology (including renal maturation and bone remodelling); (ii) multiple dosing or long-term exposure; (iii) additional routes of exposure such as subcutaneous (s.c.) or intramuscular (i.m.) as in vaccinations or subcutaneous allergen immunotherapy (SCIT), p.o. exposure via food or antacids, and i.v. exposure from PN.

To extend the model, firstly, input kinetics of poorly soluble Al salts like Al hydroxide (AH) and Al phosphate (AP) via s.c. or i.m. administration routes, as used in SCIT and vaccine products, have to be implemented. Due to the very slow systemic release of Al in these settings, plasma concentration data are unsuitable for estimation of s.c. or i.m. absorption rates (Weisser et al. 2019, 2020). As an alternative approach, adjuvant-specific rates of Al loss from the injection site can be used, assuming that they translate to Al absorption into blood. For this, our recent studies in rats can be used, together with published studies on absorption site kinetics in animals treated s.c./i.m. with Al-adjuvanted products (Weisser et al. 2019, 2020; Flarend et al. 1997; McDougall et al. 2016; Verdier et al. 2005).

Secondly, for these medicinal products as well as for PN solutions, applications in younger age groups including infants and children are particularly relevant. Curated physiological and anatomical reference values have been assembled, for adults as well as children (ICRP 2002). Being physiologically parametrised, the $\textrm{Al}$ modelling approach in Hethey et al. (2021) is able to leverage such data for population-specific $\textrm{Al}$ exposure simulations. Importantly, due to the slow absorption of Al from poorly soluble Al adjuvants (Weisser et al. 2019, 2020) and multiple applications over months (vaccinations) or years (SCIT), as well as slow Al release kinetics from bone on the timescale of years, simulations need to cover a long period of time during which physiology may change markedly, especially during infancy or adolescence. Therefore, the model should be able to dynamically adjust physiological parameters by age. As renal elimination is the predominant elimination pathway of Al from plasma (Yokel and McNamara 2001), it is important to also account for maturation of kidney function (increase of glomerular filtration rate) during infancy and childhood.

Lastly, since bone is a major organ of Al accumulation (Kruger et al. 2014; Weisser et al. 2019, 2020), alterations in bone turnover during childhood and adolescence need to be accounted for. Indeed, Al belongs to the bone-volume seeking elements, for which the kinetic behaviour is largely determined by the balance among calcium (Ca) excretion, uptake and release from bone (O’Flaherty 1991a). Consequently, several authors have formulated mechanistic models for other bone-volume seeking elements (lead and strontium) on the premise that their uptake into and release from bone parallels that of Ca (Leggett 1992; Richardson 2010; Pertinez et al. 2013; O’Flaherty 1993). We have recently established age-dependent Ca kinetic rates in Caucasians based on a comprehensive literature review (Hartung et al. 2024). These rates are well suited to form the basis for a bone module accounting for the major changes in bone metabolism in childhood and adolescence.

With an extended model scope, a thorough validation is required, including the additional routes of administration, multiple dosing and long term exposure, different age groups (infants/children/adults), and human organ data (beyond blood/urine). Since kinetic data using $^{26}\textrm{Al}$ have been exhausted during the initial model development (Hethey et al. 2021) and are more limited in scope, $^{27}\textrm{Al}$ data needed to be exploited. However, in contrast to the tracer $^{26}\textrm{Al}$, which can be quantified in low amounts since it doesn’t occur naturally, background exposure (from food and at birth) needs to be taken into account when considering $^{27}\textrm{Al}$ data. A number of different $^{27}\textrm{Al}$ exposure scenarios can be leveraged, including in particular (i) reference Al tissue concentrations in adults from autopsy studies; (ii) elevated Al exposure scenarios in children and adults (e.g. Al-containing PN solutions); and (iii) data from adjuvant exposure in animals and humans. We have compiled a comprehensive set of validation data from the literature, focussing on bone as the toxicologically most relevant organ but also considering plasma, liver, brain, and other tissues.

This article presents the details of the extension of the Al PBTK model from Hethey et al. (2021) regarding all these aspects and its thorough validation, thereby making it ready for use in risk assessment, e.g. of vaccines and immunotherapeutics.

## Methods

### Structure of the PBTK model

#### Core model structure

The core model structure of our Al PBTK model is identical to our previous model from Hethey et al. (2021). It is comprised of ordinary differential equations (ODEs) describing the rate of change of Al in blood and tissues (bone, brain, liver, kidney, spleen, muscle, rest of body), combined with dosing compartments and an elimination compartment (urine), with a physiological parametrization typical for PBTK models (Jones and Rowland-Yeo 2013). In blood, two different Al species are modelled, namely “Mix” (equilibrium of Al salts) and “addCit” (excess Al citrate), with a first-order transfer term describing equilibration of Al salts after i.v. dosing of Al citrate. Distribution into tissue is modelled without distinguishing between Al species, due to a lack of sufficient quantitative understanding of the uptake mechanisms. $\textrm{Al}$ redistribution from tissue to blood is divided into addCit and Mix according to the current addCit:Mix ratio. For all tissues except bone, the uptake and release rate constants are parametrised as follows:

1 $$\begin{aligned} k_\mathrm {{\textrm{blo}}2{\textrm{tis}}} = \frac{I_\textrm{tis}\cdot Q_\textrm{tis}}{V_\textrm{blo}}, \qquad k_\mathrm {{\textrm{tis}}2{\textrm{blo}}} = \frac{Q_\textrm{tis}}{ K_\textrm{tis}\cdot V_\textrm{tis}}, \end{aligned}$$

with tissue blood flow $Q_\textrm{tis}$, total blood volume $V_\textrm{blo}$ and tissue volume $V_\textrm{tis}$ being physiological parameters and the uptake and retention coefficients $I_\textrm{tis}$ and $K_\textrm{tis}$ estimated parameters, with $I_\textrm{tis}$ empirically modelling tissue uptake processes not fully understood mechanistically.

The data basis for parameter estimation was a comprehensive collection of $^{26}\textrm{Al}$ tracer studies from the literature (Weisser et al. 2017; Hethey et al. 2021), focussing on rats and humans and Al chloride and Al citrate as the most frequently studied species and (soluble) Al salts, respectively. The choice of tissues was also based on data availability. Physiological and estimated parameters were assumed to be tissue-specific, except that visceral organs (liver, spleen, kidney) were assumed to share uptake and retention coefficients (due to common physiological properties and data sparsity). More details on the above processes and assumptions can be found in Hethey et al. (2021).

**Fig. 1:**
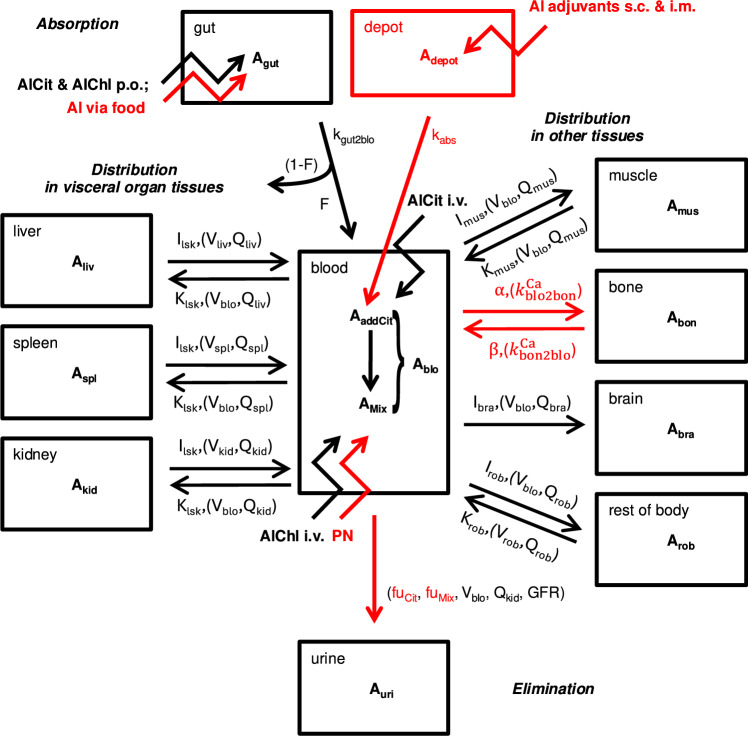
Structure of the Al PBTK model. Each compartment represents an ordinary differential equation, straight arrows indicate an exchange of $\textrm{Al}$ between compartments and jagged arrows are dosing sites. All exchange processes were modelled as first-order, except that absorption of Al adjuvants was modelled as a zero-order process. Symbols written on top of arrows are model parameters, with (non-estimated) physiological parameters in parentheses. Modules/parametrizations which have changed or are newly added compared to Hethey et al. (2021) are highlighted in red. Abbreviations: AlCit, aluminium citrate; AlChl, aluminium chloride; PN, parenteral nutrition; i.v., intravenous; p.o., per oral; s.c., subcutaneous; i.m. intramuscular

The newly implemented modules for distribution into bone and renal clearance, the implementation of various dosing routes and physiological growth are discussed in separate sections below. The extended model structure is illustrated in Fig. 1, also indicating updates with respect to Hethey et al. (2021). The full system of ODEs is provided in the Supplementary Material, Sect. S1.

#### Dynamic bone module based on calcium kinetics

In our previous model (Hethey et al. 2021), bone uptake and release rates were parametrised using Eq. 1, like any other organ. While this parametrisation reflected the effects of net bone growth, it did not capture the known changes in bone turnover during childhood and adolescence.

Similarly to other models for bone-seeking elements like lead (O’Flaherty 1993), and strontium (Pertinez et al. 2013), we therefore linked $\textrm{Al}$ uptake into and release from bone to that of Ca, thereby leveraging the detailed knowledge on changes in Ca kinetics during growth. We recently developed a consolidated age- and sex-dependent description of Ca deposition and release rates ($v^\text {Ca}_{+}$ and $v^\text {Ca}_{-}$, respectively) into bone in humans, which is moreover consistent with the concurrent changes in body size (weight, bone mass) (Hartung et al. 2024). To be usable in the Al PBTK model, these rates first needed to be transformed into first-order uptake and release constants via

$$\begin{aligned} k_\text {bon2blo}^\text {Ca} = \frac{v^\text {Ca}_{+}}{V_{\textrm{blo}}\cdot C^\text {Ca}_\textrm{blo}};\qquad k_\text {blo2bon}^\text {Ca} = \frac{v^\text {Ca}_{-}}{V_{\textrm{bon}}\cdot C^\text {Ca}_\textrm{bon}}. \end{aligned}$$

Ca concentration in human blood $C^\text {Ca}_\textrm{blo}$ is stable over the lifetime, and was fixed was fixed to 2.5 mmol/L based on literature (Sofronescu 2019), while Ca concentration in bone was determined based on bone calcium content and bone density, i.e. $C^\text {Ca}_\textrm{bon}= f_\text {Ca}\cdot D_\text {bon}$, from ICRP (2002). In rats, we assumed $C^\text {Ca}_\textrm{blo}$ = 2.25 mmol/L (Granjon et al. 2016) and derived Ca uptake and release constants $v^\text {Ca}_{+}$ and $v^\text {Ca}_{-}$, as well as calcium concentration in bone $C^\text {Ca}_\textrm{bon}$, from a rat bone growth model proposed in O’Flaherty (1991a, 1991b). Details are given in Supplementary Material section S2.

Then, first-order $\textrm{Al}$ uptake and release constants were determined via

$$\begin{aligned} k_\text {blo2bon}^\text {Al} = \alpha \cdot k_\text {blo2bon}^\text {Ca};\qquad k_\text {bon2blo}^\text {Al} = \beta \cdot k_\text {bon2blo}^\text {Ca}, \end{aligned}$$

with species-independent $\alpha$ and $\beta$. Since mechanistically, release of Ca and Al from bone are both assumed to be due to bone resorption by osteoclasts, the typical value of $\beta$ was fixed to 1, meaning that the typical Al release rate is equal to the reference release rate determined for Ca (however, random inter-individual variability around this value was included). Al uptake from blood to bone is assumed proportional to that of Ca (and not equal) since uptake mechanisms differ, similarly to other TK models for bone-seeking elements (e.g. Leggett (1992), Assumption1). In total, this bone module contained three parameters (fixed+random effects for $\alpha$ and random effect for $\beta$), compared to four in the previous model version (fixed+random effects for bone uptake and retention coefficients).

#### Renal clearance module

Al is almost exclusively excreted via the kidneys, mainly as Al citrate (Priest 2004; Shirley and Lote 2005). The previous model in Hethey et al. (2021) featured an effective renal clearance (CL), which accounted for the Al citrate fraction in blood. While not being a problem for single doses as considered in Hethey et al. (2021), this formulation proved to be inappropriate for combined dosing schedules (e.g. p.o. and i.v. routes in parallel), where it led to implausible (non-additive) interactions. We therefore replaced the effective CL by separate Cit and Mix clearances,

$$\begin{aligned} \text {CL}_\text {Cit} = \frac{Q_\text {kid}\cdot \text {fu}_\text {Cit}\cdot \text {GFR}}{Q_\text {kid}+\text {fu}_\text {Cit}\cdot \text {GFR}}, \qquad \text {CL}_\text {Mix} = \frac{Q_\text {kid}\cdot \text {fu}_\text {Mix}\cdot \text {GFR}}{Q_\text {kid}+\text {fu}_\text {Mix}\cdot \text {GFR}}, \end{aligned}$$

with the same ultrafiltrable fractions $\text {fu}_\text {Cit} = 1$ and $\text {fu}_\text {Mix} = 0.1$ as in (Hethey et al. 2021), and GFR derived from species-specific models as explained in section 2.1.5. During model development in Hethey et al. (2021), estimation issues were encountered with such a model, but together with other reformulations, a successful estimation was now possible (see Sect. 3.1).

#### Dosing module

The previous model in Hethey et al. (2021) featured i.v. bolus dosing of Al citrate and chloride (into compartments “addCit” and “Mix”, respectively), as well as p.o. dosing, with a common absorption rate and bioavailability for the two salts and assuming systemic uptake via compartment “Mix”. We extended the dosing module to include other routes as well:

- Al exposure via food was modelled using the same bioavailability and absorption rate as for p.o. dosing, but as a continuous uptake rate rather than discrete (meal) events;
- For orally dosed Al-based antacids, we used the reported bioavailability for the specific antacid, together with the estimated absorption rate for soluble Al salts;
- Al exposure through PN was treated the same way as i.v. dosing of Al chloride, since PN solutions are expected to mainly contain this Al salt;
- s.c. and i.m. dosing of Al adjuvants was modelled using zero-order release kinetics from a depot compartment as Al citrate. To this end, injection site release data after s.c. and i.m. administration of different Al adjuvants (Al hydroxide (AH) or Al phosphate (AP), either prepared in situ or as commercial gel formulations (e.g. Alhydrogel, Adju-Phos)) in various animal species (rat, rabbit, monkey) were collected from literature (Flarend et al. 1997; McDougall et al. 2016; Verdier et al. 2005; Weisser et al. 2019, 2020). Based on these data, zero-order absorption constants were estimated for each adjuvant, based on a linear regression with fixed intercept at 100%. These absorption kinetics were used for simulations of Al-containing adjuvants in both rats and humans.

#### Age-dependent physiology

In Hethey et al. (2021), all considered human physiological and anatomical parameter values were from adults and assumed as static. For children, however, age-dependent physiological changes during Al exposure need to be accounted for, especially when considering slow processes such as Al release from bone. We therefore rendered all physiological and anatomical parameters age-dependent, from (term) newborns to adults, as explained below. For most parameters, literature values for different age groups (newborn, 1 year, 5 years, 10 years, 15 years and adult) were linearly interpolated: body weight, organ weights and bone density were taken from ICRP (2002), and blood flows were derived using a lean body weight/fat mass-based scaling approach proposed in Huisinga et al. (2012). Of note, the definition of the bone compartment was changed compared to Hethey et al. (2021), now representing cartilage-free bone instead of total bone comprising cartilage (see Sec. S3 for details). For Ca-related parameters and GFR, specific models were used. Ca bone deposition and release rates $v_{+}^\text {Ca}$ and $v_{-}^\text {Ca}$ were chosen as explained in Sec. 2.1.2. GFR (in mL/min) was calculated based on an allometric scaling/maturation approach described in Rhodin et al. (2009),

2 $$\begin{aligned} \text {GFR} = \text {GFR}_\text {ref} \cdot \left( \frac{\text {LBW}}{\text {LBW}_\text {ref}}\right) ^{0.632} \cdot \frac{\text {PMA}^{3.33}}{\text {PMA}^{3.33} + \text {TM}_{50}^{3.33}} \end{aligned}$$

where lean body weight $\text {LBW}$ is predicted based on body weight and height using a model by Janmahasatian et al. (2005), PMA is post-menstrual age (age from birth plus a pregnancy duration of 0.77 y), and with parameters $\text {GFR}_\text {ref} = {112}\,\mathrm{mL/min}$, $\text {LBW}_\text {ref} = {56.1}\,\textrm{kg}$, and $\text {TM}_{50} = {1.06}\,\textrm{y}$, the latter being the maturation half-time. The age-dependency of all physiological parameters is illustrated in Fig. S4.

For rats, static physiologies were used, as in Hethey et al. (2021). For each simulation, the rat body weight was chosen to be either 250 g or 475 g, with associated detailed physiologies reported in Hethey et al. (2021). For studies with short duration ($\le$5 d), the reference rat with the smallest difference to the mean body weight at study onset was chosen. In case of longer study duration, the number of data points generated at time points $\le$5 d and >5d were counted. If the majority of data points were $\le$5d, the starting weight of the rats were used for allocation. Otherwise, the heavy reference rat (475 g) was used, since rats are growing rapidly and reach their adult weight within a few weeks (e.g. a 300 g Wistar rat has grown to about 400–450 g within 30 days, Janvier (2024)). Rat GFR was predicted based on body weight (BW) using the same approach as described in Hethey et al. (2021), namely a purely allometric relationship

3 $$\begin{aligned} \text {GFR} = \text {GFR}_\text {ref} \cdot \left( \frac{\text {BW}}{\text {BW}_\text {ref}}\right) ^{0.75} \end{aligned}$$

with $\text {GFR}_\text {ref} = {0.0786} \,\mathrm{L/h}$ reported by Davies and Morris (1993) for a rat with body weight $\text {BW}_\text {ref} = {250}\,\textrm{g}$.

### Simulation details

All numerical simulation results were obtained using **R** version 4.2.2 (R Core Team 2022), using package mlxR version 4.2.0 (Lavielle 2021) for simulation of the Al PBTK model. For all exposure scenarios, $n=500$ individuals were simulated and simulation results displayed as median and 90% confidence intervals. Simulations compared to individual data were always matched by age and sex. In contrast, for simulations compared to summary statistics, a mixed male/female population was simulated and the average reported age used. R Markdown scripts to produced all results are available on Zenodo (https://doi.org/10.5281/zenodo.14710873).

#### Initial conditions

Especially for children below 1 year of age, initial Al levels built up during embryonal development need to be considered. To this end, the literature was screened for reports on normal Al concentrations in healthy newborns. Six references were identified, reporting on one or several organs amongst plasma, bone, brain, liver and kidney (Moreno et al. 1994; Bozynski et al. 1989; Sedman et al. 1985; Litov et al. 1989; Bougle et al. 1992; Hawkins et al. 1994). These reported values were used to identify the parameters of an assumed underlying lognormal distribution of Al concentrations (for details, see Section S6.1). Separately for each organ, lognormal distributions obtained this way from different literature sources were averaged arithmetically; subsequently, another lognormal distribution was fitted to the averaged distribution, yielding the final distribution from which to sample initial values. This process is shown in Fig. S3. For muscle and spleen, no literature values on Al concentrations in newborns could be retrieved. Spleen being a visceral organ like liver and kidney and the Al concentration distributions for the latter two organs being very similar, an average of the liver and kidney distributions profiles was assumed for spleen (consistent with the use of common intrusion and retention coefficients $I_\textrm{lsk},K_\textrm{lsk}$ in visceral organs in the Al PBTK model, leading to identical steady-state concentrations). For muscle as well as for rest of body, both of which are in quick exchange with plasma, a quasi-steady state with plasma was assumed, based on the parameter estimates reported in Table S2.

We found no data on the correlation structure of initial values in different organs. A plausible assumption is that if the initial value is large in one organ, this is also the case in other organs. We used a simple mathematical representation thereof, assuming the distributions in different organs to be perfectly correlated. Algorithmically, this means that, for each simulation, a single standard normally distributed value was drawn, which was then scaled to match mean and variance of each normal distribution underlying the lognormal initial distributions in different tissues, and exponentiated.

For consistency, these initial value distributions were applied for all simulations of $^{27}\textrm{Al}$-based studies in humans (children and adults), except for Klein et al. (1982); Stoehr et al. (2006) which start at adult age, neglecting baseline exposure due to the long duration and large exposure of the PN solution.

#### Reference Al intake via food

For simulation scenarios of $^{27}\textrm{Al}$ exposure, the underlying intake by food (depending on age) needs to be included, except during PN. An average realistic Al intake via food used in the simulations was taken from a review of European studies published by EFSA (2008); see Table 1 for an overview and Supplementary Material section S5 for details.

**Table 1:** Average Al intake via food based on European studies on food consumption (EFSA 2008), which was used in the simulations

Age	0–3 mo	3–6 mo	6–9 mo	9–12 mo	12 mo–adult
Intake [mg Al/kg BW/week]	0.1	0.2	0.4	0.8	0.8

Since the estimated bioavailability (F) of 0.168% in our model (see Table S2), representing an average for Al chloride and Al citrate in solution, was in good agreement with commonly accepted values of 0.1% (food) – 0.3% (drinking water) for Al absorption from diet (Tietz et al. 2019; Yokel and McNamara 2001), this estimate was used for exposure from food as well.

#### Dry versus wet tissue weight

The content of Al measured in bone and other tissues is usually reported as $\mu$g/g dry weight or wet weight, depending on the condition of the sample when it was weighed. In the Al PBTK model, all organs including bone are considered as “wet”. Therefore, all literature data reported as dry weight were transformed to wet weight to allow for a meaningful comparison. In addition, bone samples were considered as marrow-free bone tissue (which was mostly, but not always clearly indicated), whereas the model bone compartment comprises cartilage-free skeleton including bone marrow, which also needed to be accounted for when transforming the data. Conversion factors to derive wet weight (model) concentrations from dry weight data in humans are given in Table 2; their derivation from literature values can be found in Supplementary Material section S4.

**Table 2:** Dry-to-wet weight conversion factors (*C*), such that 1 $\upmu \textrm{g}/\textrm{g}$ dry weight $\hat{=}~C$ $\upmu \textrm{g}/\textrm{g}$ wet weight

	Bone	Brain	Kidney	Liver
Newborn–10 $\textrm{y}$	0.566	(assumed identical to adults)		
Adult	0.497	0.23	0.22	0.25

### Literature search and summary of validation datasets

The literature was screened for $\textrm{Al}$ concentration data under diverse conditions to evaluate the model’s capacity to correctly predict $\textrm{Al}$ exposure. In contrast to our previously assembled $^{26}\textrm{Al}$ dataset in Hethey et al. (2021), here we also considered $^{27}\textrm{Al}$ data to allow for a more thorough validation. In total, 22 references were selected for model validation (see Table 3), as explained below.

Normal $^{27}\textrm{Al}$ plasma and tissue concentrations in healthy adults allow for an evaluation of the model’s capacity to correctly predict the impact of long-term $\textrm{Al}$ exposure, presumably mainly via food. Only studies explicitly designed to collect normal values (not control groups in non-representative populations such as hospitalized patients) were considered. Reference published more than 30 years ago were excluded due to potential changes in $\textrm{Al}$ exposure over time. We identified 11 references reporting reference $^{27}\textrm{Al}$ concentrations in different tissues in adults. Besides in plasma and toxicologically relevant organs (bone, brain and liver), we also found reference values in kidney, spleen and muscle. These values were collected from both US/Canadian (Krewski et al. 2007; Tang et al. 1999; Kruger et al. 2014; McLachlan et al. 2018) and European populations (Hellström et al. 2005, 2006; House et al. 2012; Exley and Clarkson 2020; Roider and Drasch 1999; Rahil-Khazen et al. 2002; Hongve et al. 1996). Two studies reported results based on wet tissue weight (Roider and Drasch 1999; Rahil-Khazen et al. 2002), the other nine studies in terms of dry tissue weight. The latter were converted to wet weight as described in Sect. 2.2.3.

PN, and especially total (i.e. exclusive) PN, may result in elevated $^{27}\textrm{Al}$ exposure. To identify suitable validation datasets, we performed a literature search in Pubmed and Google scholar using the search terms “aluminium” AND “parenteral nutrition”. The results were kept if they contained data on both Al measurements in plasma or tissues during/directly after PN and the cumulative PN volume administered up to the sampling timepoint(s) including Al content, allowing calculation of the total Al intake during PN. Furthermore, individuals had to be on PN for at least 90% of their lifespan without any other (enteral) source of Al, or with such a high Al exposure that any (unknown) baseline exposure could likely be neglected. This literature search yielded six validation studies. Four of these reported on $^{27}\textrm{Al}$ exposure in plasma and tissues in newborns and young children under total PN (Klein et al. 1984; Moreno et al. 1994; Advenier et al. 2003; Courtney-Martin et al. 2014). The study by Klein et al. (1982) reported on adults; it was included since it contained measurements in bone and had a high Al exposure via PN (with osteomalacia symptoms). One further study reported on a combined PN and antacid treatment in adults (Stoehr et al. 2006), which was used to test the model in a scenario with simultaneous elevated Al exposure via the p.o. and i.v. routes.

Predicting exposure of adjuvanted $^{27}\textrm{Al}$ in immunotherapy or vaccine products is of high regulatory relevance. Recently, three studies on TK of Al-containing adjuvants have been published, representing a major opportunity for evaluation of our Al PBTK model in this context. Weisser et al. (2019, 2020) reported on elevated bone Al levels after single dose administrations of different adjuvant formulations in rats (s.c. or i.m., respectively). In addition, Hiller et al. (2024) found elevated urinary Al excretion rates after long-term Al-based SCIT (1.13 mg Al, 74 $\times$ every 5 weeks) in 11 humans with normal renal function, compared to a control group before initiation of SCIT. Data, reported as $\upmu \mathrm{g Al/g}$ creatinine, were transformed to $\upmu \mathrm{g Al/d}$ as described in Sect. S6.2. We integrated these three studies on adjuvanted $^{27}\textrm{Al}$ as validation data.

Finally, the $^{26}\textrm{Al}$ retention datasets by Newton and Talbot (2012); Talbot et al. (1995), already used as validation data in our previous study (Hethey et al. 2021), were again considered. In particular, the long-term $^{26}\textrm{Al}$ retention by Newton and Talbot (2012) allowed to evaluate model predictions for Al accumulation in human bone, the major organ of long-term Al storage.

Data were directly extracted from text and tables or digitised from figures using WebPlotDigitizer (Rohatgi 2022). Individual data for Courtney-Martin et al. (2014) were obtained by contacting the authors.

**Table 3:** Overview on validation data. Validation results for studies/tissues *in italics* are given in the supplementary material

Al exposure	Population	Route	Tissues in which Al was measured									$n_\text {ID}$	Reference
Food only (presumably)	Adults	p.o.	pla									–	(Krewski et al. 2007)
	Adults	p.o.		bon								5	(Tang et al. 1999)
	Adults	p.o.		bon								69	(Hellström et al. 2005)
	Adults	p.o.		bon								62	(Hellström et al. 2006)
	Adults	p.o.		bon								18	(Kruger et al. 2014)
	Adults	p.o.			bra							60	(House et al. 2012)
	Adults	p.o.			bra							75	(McLachlan et al. 2018)
	Adults	p.o.			bra							20	(Exley and Clarkson 2020)
	Adults	p.o.			bra	liv	*kid*	*spl*				136–149$^{2}$	(Roider and Drasch 1999)
	Adults	p.o.				liv	*kid*	*spl*	*mus*			24–30$^{2}$	(Rahil-Khazen et al. 2002)
	Adults	p.o.				liv						95$^{3}$	(Hongve et al. 1996)
Adjuvants	Rats	i.m.	*pla*	bon	bra							33$^{4}$	(Weisser et al. 2019)
	Rats	s.c.	*pla*	bon	bra							18$^{4}$	(Weisser et al. 2020)
	Adults	s.c.								uri		11	(Hiller et al. 2024)
PN	Children	i.v.	*pla*			liv						5	(Klein et al. 1984)
	Neonate	i.v.		bon	bra	liv	kid					1	(Moreno et al. 1994)
	Children$^{1}$	i.v.	*pla*							*uri*		4	(Advenier et al. 2003)
	Children$^{1}$	i.v.	*pla*									3	(Courtney-Martin et al. 2014)
	Adults	i.v.	*pla*	bon								6	(Klein et al. 1982)
PN+antacid	Adults	i.v.+p.o.	pla							uri		15	(Stoehr et al. 2006)
$^{26}\textrm{Al}$ injection	Adults	i.v.									ret	5	(Talbot et al. 1995)
	Adults	i.v.									ret	1	(Newton and Talbot 2012)

Abbreviations: PN, parenteral nutrition; $n_\text {ID}$, number of individuals (per tissue); pla, plasma; bon, bone; bra, brain; liv, liver; kid, kidney; spl, spleen; mus, muscle; uri, urine; ret, whole-body retention. 1: some individuals were excluded since they received PN for less than 90% of their life. 2: range of $n_\text {ID}$ reported for different tissues. 3: number of liver samples (number of individuals not specified). 4: total number of treated animals with different adjuvants, excluding controls

## Results

### Estimation results

After extending the model from Hethey et al. (2021) as described in the Methods, model parameters were re-estimated on the previously described $^{26}\textrm{Al}$ dataset (Hethey et al. 2021) with the bone data by Ittel et al. (1997) corrected for dry vs. wet weight (see Supplementary Material section S4). Upon the above changes, an estimated value of parameter $\text {K}_\text {rob}$ improved the model fit compared to fixing it to 1 as in the previous model version. Estimation results are reported in Table S2. Compared to the Hethey et al. (2021) model, goodness-of-fit was essentially unchanged (see Figs. S1 and S2), demonstrating that the additional model features and/or modules did not compromise estimation performance.

In contrast to our previous model in Hethey et al. (2021), $\textrm{Al}$ exposure can now also be predicted in children, where an increased calcium turnover leads to larger rates of uptake into and release from bone, compared to adults.

### Model validation: Al exposure via enteral food uptake

To validate the model performance in a multiple dosing scenario, we first simulated repeated p.o. ingestions of Al by mimicking the average dietary Al intake in Europe over time from birth to adulthood (see Table 1), using a simulation window of 50 years. Concentrations of Al in plasma and tissues were predicted and compared to reference values in literature (see Table 3).

**Fig. 2:**
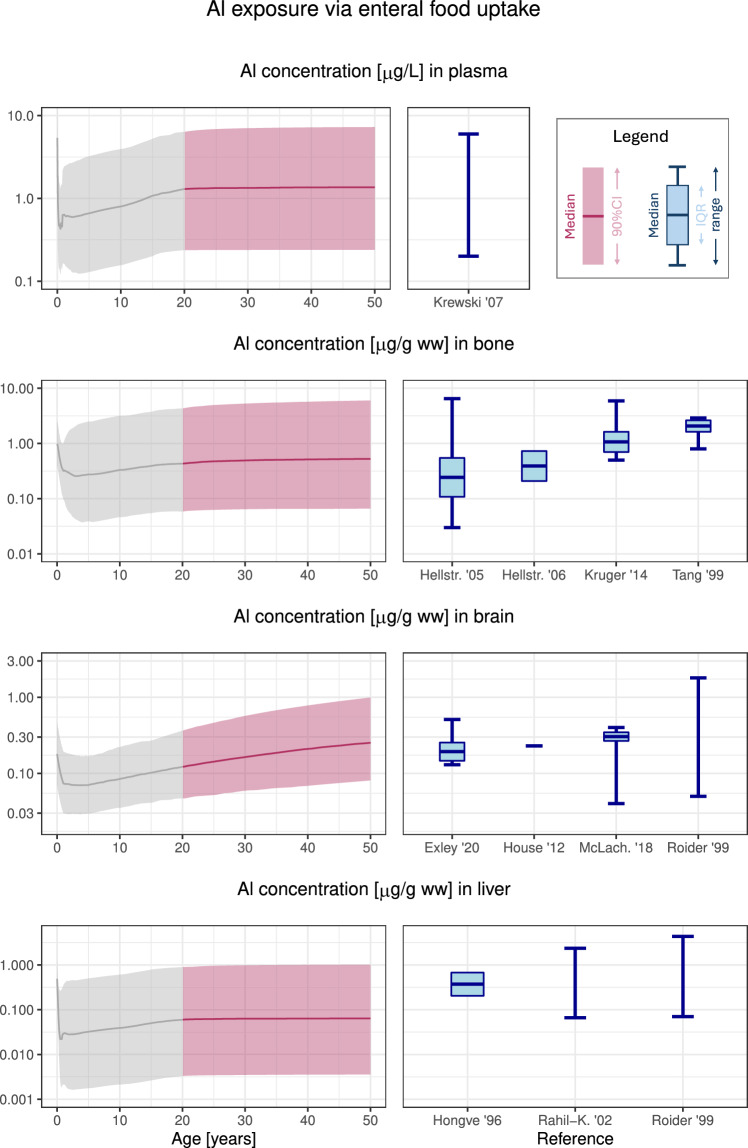
Reference Al concentrations in adults from enteral uptake via food according to simulations (left, adult range 20–50 years highlighted) and from literature data (right). (Calculated) median, interquartile range and/or range are displayed as reported in the respective literature source. Abbreviations: ww, wet weight; CI, confidence interval; IQR, interquartile range; Hellstr., Hellstroem; McLach., McLachlan; Rahil-K., Rahil-Khazen

Results for plasma and the toxicologically relevant organs bone, brain and liver are shown in Fig. 2. Plasma, bone and brain reference levels fitted very well to the prediction by the Al PBTK model, while levels reported in liver were slightly higher than, but still compatible with the predictions. For all organs, the range of variability was well captured in the Al PBTK model. Considering the exposure from food to be appropriately represented in the model, we proceeded to other evaluation scenarios.

### Model validation: subcutaneous and intramuscular exposure of Al from adjuvants

**Fig. 3:**
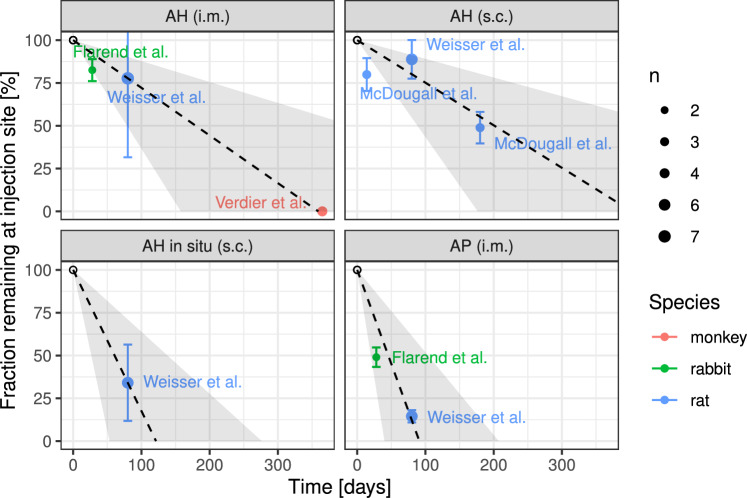
Fraction of injected Al amount remaining at the injection site (mean±standard deviation from *n* samples) over time from various animal studies after subcutaneous (s.c.) or intramuscular (i.m.) injection of products adjuvanted with different Al preparations. Dashed line (shaded area): predicted fraction remaining (90% confidence intervals) using the estimated adjuvant-specific zero-order absorption rates. Abbreviations: AH, Al hydroxide; AP, Al phosphate

We next validated model predictions for $\textrm{Al}$ exposure from adjuvanted SCIT and vaccine products. To this end, we first estimated zero-order absorption rates $k_\text {abs}$ for each of four adjuvant types and routes (AP i.m.: 0.01098/day, AH i.m.: 0.002784/day, AH s.c.: 0.0024864/day, AH in situ s.c.: 0.0082392/day). The injection site release data (from rats, monkeys and rabbits) used for estimation and the resulting release kinetics are shown in Fig. 3. Since these absorption rates are expected to differ between individuals, we assumed them to be lognormal distributed in the population, with a 50% coefficient of variation across products. This variability estimate seems plausible when considering the variability in Fig. 3 and it is comparable to that of other s.c. administered compounds such as monoclonal antibodies (Dirks and Meibohm (2010), Table II).

**Fig. 4:**
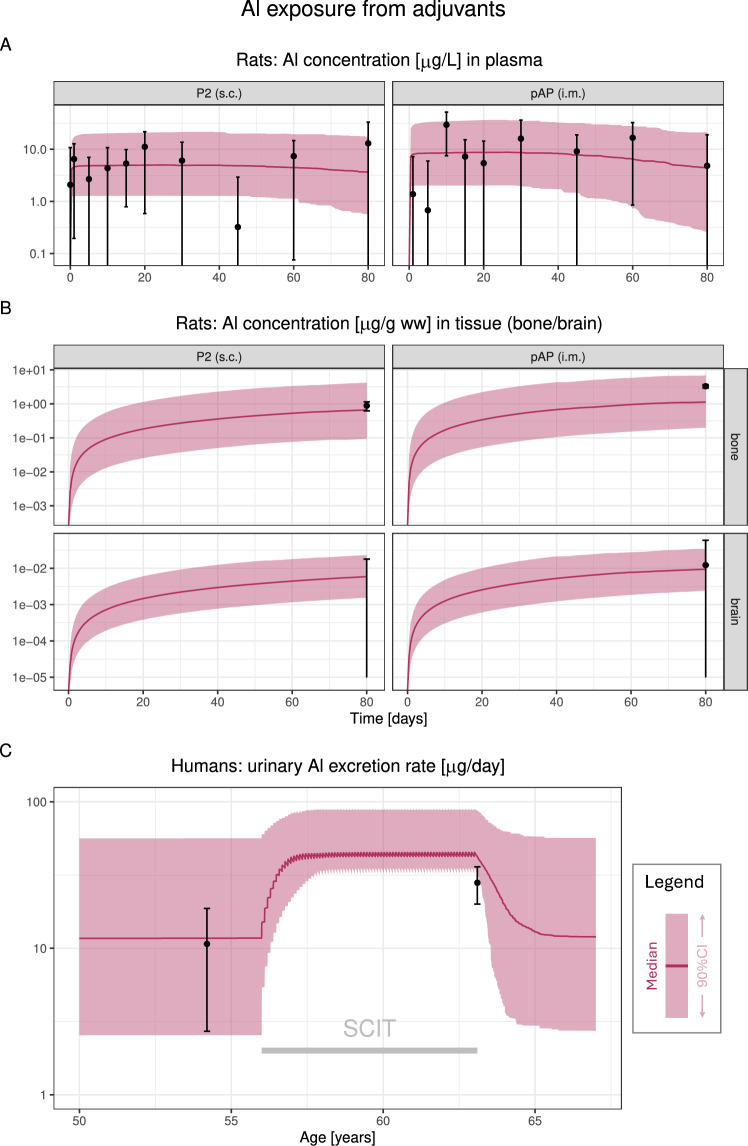
Model validation for Al exposure from adjuvants in rats (**A**, **B**) and humans (**C**). **A**/**B**: comparison of the model predictions to experimental data for two Al-containing adjuvants in rat plasma (**A**) and tissues (bone and brain, **B**). Simulated Al exposure is via adjuvants only, and data represent excess Al exposure compared to a control group, displayed as mean±SD (note that mean−SD or mean can become negative this way, and whiskers extend to the bottom plot range in this case). Simulations for other adjuvants can be found in the Supplementary Material (Fig. S6). **C**: comparison of the model predictions to measured urinary Al excretion rates in humans on subcutaneous immunotherapy vs. control (enteral uptake from food only). Abbreviations: pAP (i.m.), intramuscular administration of Al phosphate-containing Adjuphos; P2 (s.c.), subcutaneous administration of Al hydroxide-containing allergen product no. 2; SCIT, subcutaneous immunotherapy; CI, confidence interval

Using these absorption rates, we simulated Al exposure in rats for the different adjuvants described in Weisser et al. (2019, 2020), as summarised in Table S3. Since no module for initial conditions and exposure via food was available for rats, we modelled the increase of Al concentrations in treatment vs. control groups (for details, see Section S6.3), using the Al PBTK model with zero initial conditions and without baseline exposure from food. For two of the treatments, measured $\textrm{Al}$ concentrations were significantly increased in plasma and bone, but not in brain. The model predictions accurately captured both the extent of increase in plasma and bone, as well as a negligible increase in brain exposure (see Fig. 4AB).

Applying the adjuvant-specific absorption rate, we simulated Al exposure via food combined with an AH-containing SCIT in humans according to Hiller et al. (2024). The model predictions were in good agreement with the reported Al urinary excretion data before and during SCIT (see Fig. 4C).

### Model validation: Al exposure from parenteral nutrition

Next, we considered three studies reporting elevated Al exposure during Al-containing PN in a newborn (Moreno et al. 1994), a population of children aged 18 to 34 months (Klein et al. 1984) and a population of adults with normal renal function receiving PN for 1–6 years (Klein et al. 1982).

**Fig. 5:**
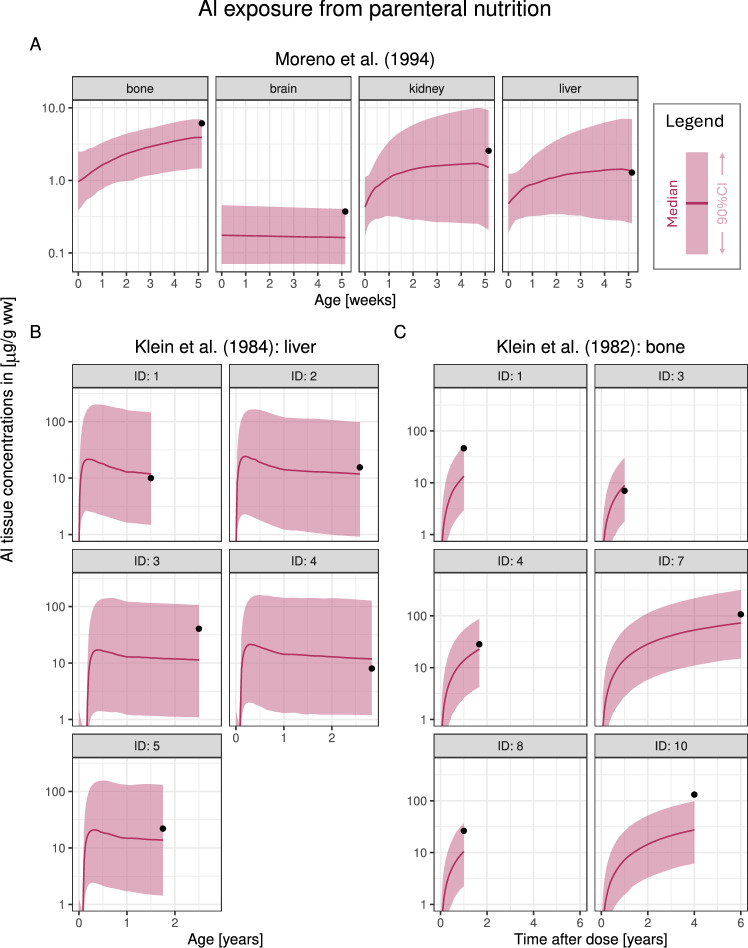
Model validation using Al tissue data obtained in individuals on total parenteral nutrition (PN). **A**: one term newborn (Moreno et al. 1994), **B**: a population of children aged 18 to 34 months (Klein et al. 1984), **C**: a population of adults receiving PN for 1–6 years (Klein et al. 1982). Simulations were stopped at the timepoint of sampling. Abbreviations: ww, wet weight; CI, confidence interval

Moreno et al. (1994) reported Al concentrations in bone, brain, kidney and liver in a term newborn receiving total PN for 33 days, demised 3 days after. The Al PBTK model accurately predicted the largely increased bone concentration as well as elevated exposure in kidney and liver; the observed brain concentration was explained by the initial Al concentrations at birth and not the short-term Al exposure via PN (see Fig. 5A).

Next, Klein et al. (1984) reported on fiv children aged 8 to 34 months who had received total PN for 8 to 33 months, for whom Al concentrations in liver were quantified through biopsies. Elevated liver exposure were measured for all five children, which the model predicted accurately (Fig. 5B). Plasma concentrations were measured for three of these children, which the model slightly overpredicted (see Fig. S7).

Furthermore, Klein et al. (1982) reported on plasma and/or bone Al exposure in a population receiving total PN rich in Al (casein solution). Of the 11 patients reported, bone concentrations were measured in 6 (IDs 1,3,4,7,8,10), which were considered for model validation. The predicted bone exposure matched the observed data reasonably well for this patient population, for different durations and doses of PN (Fig. 5C). As for Klein et al. (1984) plasma concentrations were slightly overpredicted (see Fig. S8).

We then considered studies by Courtney-Martin et al. (2014) (plasma concentrations in three children) and Advenier et al. (2003) (plasma concentrations and urinary Al excretion rates in six children/adolescents). In contrast to the studies by Klein et al. (1982, 1984), no systematic overprediction could be seen for plasma exposure in these two studies; also predicted urinary Al excretion rates showed no sign of misfits (see Figs. S9 and S10).

**Fig. 6:**
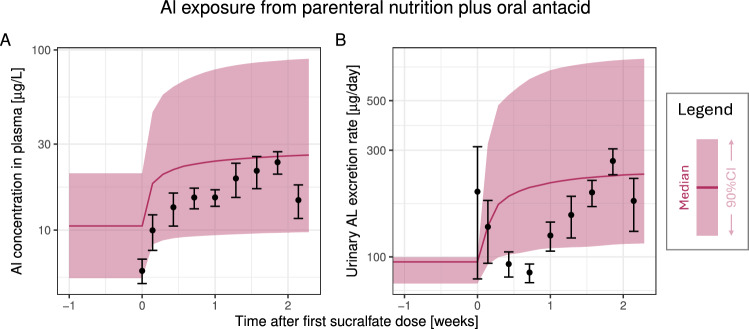
Model validation using data from a combined p.o. and i.v. exposure scenario reported by Stoehr et al. (2006). An mixed male/female adult population was simulated and compared to data (mean±SD). Prolonged parenteral Al exposure led to the model predictions reaching steady-state. Subsequently, an oral antacid (sucralfate) was added, leading to increased exposure. The first observation is a pre-sucralfate baseline observation. Abbreviations: CI, confidence interval

Finally, we considered a study by Stoehr et al. (2006), reporting on a group of 15 adult ICU patients (7 female /8 male, 2 over 60 years) who received PN and an Al-based antacid (sucralfate, Al sucrose sulphate) for stress ulcer prophylaxis (combined i.v. / p.o. administration route). Patients received a mean daily parenteral Al intake of 101.3 $\upmu \mathrm{g/d}$ (range 83–118) from various small and large volume parenterals for 45 months prior to antacid treatment. In addition, they received 1 g sucralfate six times daily by stomach tube over 15 days, which corresponds to a daily Al intake of $6\times 190 = 1140$ mg. Longitudinal Al measurements in plasma and 24 h pooled urine samples were performed. For simulation, we used their estimated sucralfate bioavailability of 0.019%. Due to the significant and prolonged parenteral Al exposure, baseline Al exposure from food prior to start of PN was deemed irrelevant and thus neglected. Both the baseline exposure via PN (time 0) and the increase in exposure due to the antacid treatment were well captured in both plasma and urine (see Fig. 6).

### Model validation: long-term $^{26}\textrm{Al}$ retention

**Fig. 7:**
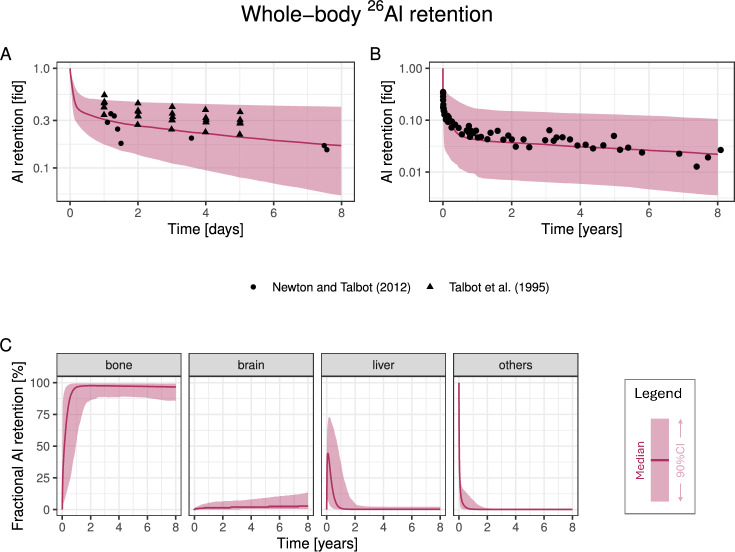
Model validation using $^{26}\textrm{Al}$ retention data after single i.v. administration as citrate. **A/B**: Comparison of fraction of retained Al in the body reported in Talbot et al. (1995); Newton and Talbot (2012), on a timescale of days (**A**) and years (**B**). **C**: simulated distribution of retained Al in different tissues. Abbreviations: fid, fraction of injected dose; CI, confidence interval

As the final validation dataset, we considered $^{26}\textrm{Al}$ whole-body retention data after a single i.v. dose of Al citrate (Talbot et al. 1995; Newton and Talbot 2012). In the first five days, the fraction of dose retained matched the model predictions well (Fig. 7A). Importantly, long-term retention (ca. 2% of the dose) was very accurately predicted in the model (Fig. 7B). Since over 90% of retained Al is in bone on the timescale of years after dosing (Fig. 7C), this dataset successfully validates our new bone module in humans.

This dataset was already considered as a validation dataset in our previous model version (Hethey et al. 2021), which used a different bone compartment parametrisation (see Methods). By decreasing predicted bone concentrations in human adults, the inclusion of calcium kinetics in the bone compartment parametrisation of the Al PBTK model presented here led to a markedly improved fit to the long-term retention data compared to Hethey et al. (2021), see Fig. S2 (middle panel).

## Discussion

We have successfully extended our previous work (Hethey et al. 2021) to arrive at an Al PBTK model we consider suitable for use in simulating many Al exposure scenarios relevant for risk assessment. By rendering physiologies age-dependent, accounting for GFR maturation and the age-dependent and Ca-informed Al turnover in humans, and including a large number of relevant uptake routes (in particular, s.c. and i.m. dosing) as well as non-zero initial values in newborns with variability, the scope of our Al PBTK model has been broadened considerably compared to Hethey et al. (2021).

The human physiologies used in all simulations were stratified by sex, and the age-adjusted dynamics incorporated the dilution effect of growth on concentration measurements (exemplified in the initial decrease Al concentrations seen in Fig. 2). While variability in GFR was explicitly accounted for in the model, other physiological parameters were set to (age- and sex-specific) reference values. Variability parameters relating to oral absorption, renal clearance and some tissue distribution processes (denoted $\omega$ in Table S2) were used to capture inter-individual differences, and the estimated GFR variability in humans was found to agree well with the analysis by Rhodin et al. (2009). The model is not applicable for preterm infants currently. Besides reference values for volumes and blood flows, also GFR values, initial concentrations in tissues and calcium-related parameters would have to be identified for preterm infants, followed by additional validations. Since such an analysis would require considerable additional effort, it was out of scope of this study.

The presented Al PBTK model has been extensively validated in diverse exposure scenarios, covering p.o. Al uptake via food, s.c. and i.m. uptake from Al-containing adjuvants, i.v. uptake from total PN, combined i.v./p.o. dosing and long-term retention after i.v. dosing. The validation dataset was very diverse and covers almost all possible modes of Al exposure, except for dermal and inhaled uptake. Heterogeneity in the data was large, and some inconsistencies were found. ID 2 in Courtney-Martin et al. (2014) had twice the Al exposure of ID 1, but the measured plasma concentration was four times less. Similarly, in Klein et al. (1984), the plasma concentration measured for ID 1 was four times less than for ID 4, at the same Al exposure and comparable liver concentrations. Lastly, in Klein et al. (1982), no correlation could be seen between individual measurements in plasma and bone. The model predictions have to be evaluated in the context of this observed variability, and improvements for individual studies or data are only warranted if they do not concur a deterioration in predictive capacity in other settings. Across all studies, the Al PBTK model does not show any systematic misfit in toxicologically relevant tissues.

To evaluate model performance against reported reference Al levels in adults, we considered food to be the most important contributor to normal Al levels and applied average intake levels reported, corresponding to 80% of the tolerable weekly intake (EFSA 2008). This intake should fit well to the reference values reported for a similar range of years (1996–2020). Al exposure from drinking water and consumer products such as antiperspirants and cosmetics appears to be negligible compared to food exposure (EFSA 2008; Becker et al. 2016; de Ligt et al. 2022). Due to uncertainties on the underlying Al exposure, we did not aim at mimicking single studies in our simulations, but rather compared the simulation of an equilibrated mixed male/female adult population with an average intake based on EFSA (2008) to several literature data sources at once. Al concentrations in plasma as well as toxicologically relevant organs (bone, brain, liver) were predicted well, both in terms of central trends and variability. Of note, our assumed intake was higher than reported in a recent German study (Tietz et al. 2019), but due to good fits to reference values and the data by Hiller et al. (2024), we considered our input scheme (together with the assumed bioavailability) realistic.

As indicated by our earlier rat studies (Weisser et al. 2019, 2020), it was confirmed in our regression analyses using data from different animal species that absorption rates differ markedly between adjuvant types. Due to data sparsity, the estimation of a non-linear input curve was not possible. Considering solubilisation of the poorly soluble Al complexes as rate-limiting, the assumption of a zero-order rate was deemed more plausible than a first-order uptake. In the rabbit study by Flarend et al. (1997), non-commercially available formulations were used. According to the protocol, i.m. AH and AP were co-precipitated (in situ), which may have led to an increased uptake, like when comparing AH in formulation to co-precipitated AH after s.c. dosing (Fig. 3). However, even if the data by Flarend et al. (1997) were excluded, the estimated absorption rates would be very similar. Extrapolation of estimated absorption rates suggest that complete absorption of Al from AH-adjuvanted allergen preparations or vaccines is expected to take about 1 year. In contrast, Al from co-precipitated AH (in situ) as well as from AP might be completed much earlier (ca. 120 days). The underlying assumption of a 100 % absorption should be considered approximative, since granuloma might be built which encapsulate residual adjuvant for an unknown time (García-Patos et al. 1995; Bergfors et al. 2014; Asín et al. 2018). The slight overestimation of Al exposure during SCIT in the simulation according to Hiller et al. (2024) could be due to such effects. Mechanistically, s.c./i.m. absorption probably occurs via more than one pathway, e.g. (i) direct absorption as solubilised Al citrate into blood vessels, and/or (ii) phagocytosis of undissolved Al complexes by immune cells. The presence of Al hydroxide crystals in macrophages after immunisation and subsequent transport into lymph nodes has been shown by Giusti et al. (2015). Al dissolution and systemic uptake via lymph flow would be a logic consequence. Our rate estimate derived from residual Al at the injection sites represents an overall absorption via all such routes.

Two of the eight Al-adjuvanted products or preparations administered s.c. or i.m. to rats in the study by Weisser et al. (2019, 2020) had increased plasma and bone concentrations compared to control animals, which we considered the most relevant data sources for model validation. Predictions in the other six products/preparations were well captured in bone as well, while a low brain exposure was predicted in all treatment groups (see Fig. S6). In three vaccine products, measured brain concentrations were slightly increased in treatment groups vs. control, whereas the Al PBTK model predicted negligible brain levels. As already discussed in Weisser et al. (2019), unusually low brain concentrations were measured in the control groups (0.08 and 0.13 $\upmu \mathrm{g/g}$). Indeed, larger values of 0.2–0.8 $\upmu \mathrm{g/g}$ are reported in the literature (Veiga et al. 2013; Lin et al. 2015), which would render the increase in brain concentration non-significant for these vaccine products. Also, the slow Al loss at injection site and low bone concentrations for the vaccine products provided evidence against elevated brain concentrations in these products. Consequently, we considered these seemingly elevated brain concentrations as false positives.

The PN validation data selection focussed on term newborns and young children who received Al from PN as the only source (almost) from birth, together with PN in adults with high exposure levels rendering any background exposure negligible. These restrictions were considered essential for validation purposes, even if only fulfilled by a limited number of studies. PN input rates were calculated from Al content in various solutions and volumes administered according to diaries, and hence bear some uncertainty as well.

Pioneering work in PBTK modelling for bone-seeking metals was conducted by Ellen O’Flaherty in the 1990s. This was the basis for our adapted bone module. A fundamental premise of this module is that Al can be informed by Ca in its movement into and out of bone, in particular the age-dependency of Al bone kinetics. For Ca kinetics, we recently compiled literature estimates into age- and sex-specific Ca deposition and release functions, both peaking during puberty (Hartung et al. 2024). As bone is a major organ of Al storage and of high toxicological relevance, the inclusion of these Ca kinetic functions constituted an essential improvement. In addition to s.c./i.m. exposure from adjuvanted Al in rat bone, our validation datasets contained human bone measurements covering very diverse exposure scenarios: short-term i.v. exposure in newborns (Moreno et al. 1994), long-term retention after short-term i.v. exposure in adults (Newton and Talbot 2012), long-term p.o. exposure in adults (food) and long-term i.v. exposure in adults with symptomatic bone disease (Klein et al. 1982). The good predictions of the Al PBTK model in all of these scenarios confirmed our bone module’s appropriateness to predict short- and long-term exposures in all ages and at both healthy and toxic levels.

Brain concentrations in human adults after long-term Al exposure (food) were also predicted accurately. For short-term exposure, baseline Al levels in brain already explained the observed concentrations in a neonate (Moreno et al. 1994). Similarly, predicted brain exposure in rats from adjuvanted Al in s.c./i.m. administered adjuvants was found to be negligible compared to controls (Weisser et al. 2019, 2020). While these observations did not contradict the model predictions, the model validation in short-term exposure scenarios in brain is less conclusive than for bone, since no studies with elevated brain concentrations compared to baseline exposure were found in literature. Of note, the report on patients with dialysis encephalopathy syndrome at severely elevated Al levels in brain by Alfrey (1978) could not be used as validation data since the Al dose is unknown.

Liver was very accurately predicted after short-term exposure via total PN in a newborn (Moreno et al. 1994) and in 5 children with hepatotoxic Al levels (Klein et al. 1984). There was as slight trend for underprediction in liver long-term exposure via food, although the observed data still fell into the predicted 90% confidence intervals.

Plasma and urine exposure were predicted well on average, but heterogeneity in the data was large. The slight overprediction of plasma concentrations for Klein et al. (1982, 1984) seems to be a specific effect for this study group, since there was no such trend in other datasets, be it PN, food or adjuvants. Remarkably, the urine data by Hiller et al. (2024), the only human data after SCIT available to date, fitted very well, confirming the appropriateness of using the s.c. absorption rates for adjuvants estimated from rat data also for humans.

Although of limited relevance for risk assessment, exposure in other tissues (kidney, spleen, muscle) was also evaluated if reported in the studies. In general, the findings for all visceral organs were similar, i.e. good prediction of short-term exposure (liver, kidney) and a slight underprediction of exposure via food (liver, kidney, spleen). Long-term exposure via food was significantly underpredicted in muscle, which might potentially be due to confounding with the rest of body compartment, which behaves similarly kinetically (both have a fast tissue uptake). More detailed data would be required for model refinements in these aspects.

To summarise, our Al PBTK model is currently the only validated model to predict Al exposure from food intake, from PN, and from adjuvanted medicinal products. It is ready for use in risk assessment of various Al exposure scenarios.

## Supplementary Information

Below is the link to the electronic supplementary material.

Supplementary file 1 (pdf 1230 KB)

## Data Availability

Data and code are available on Zenodo (10.5281/zenodo.14710873).

## References

[CR1] Advenier E, Landry C, Colomb V, Cognon C, Pradeau D, Florent M, Goulet O, Ricour C, Corriol O (2003) Aluminum Contamination of Parenteral Nutrition and Aluminum Loading in Children on Long-Term Parenteral Nutrition. Journal of Pediatric Gastroenterology and Nutrition 36(4):448–453, https://journals.lww.com/jpgn/fulltext/2003/04000/aluminum_contamination_of_parenteral_nutrition_and.5.aspx 10.1097/00005176-200304000-00005 12658033

[CR2] Alfrey AC (1978) Dialysis encephalopathy syndrome. Annu Rev Med 29(1):93–98. 10.1146/annurev.me.29.020178.000521 348053 10.1146/annurev.me.29.020178.000521

[CR3] Asín J, Molín J, Pérez M, Pinczowski P, Gimeno M, Navascués N, Muniesa A, de Blas I, Lacasta D, Fernández A, de Pablo L, Mold M, Exley C, de Andrés D, Reina R, Luján L (2018) Granulomas Following Subcutaneous Injection With Aluminum Adjuvant-Containing Products in Sheep. Vet Pathol 56(3):418–428. 10.1177/0300985818809142 30381018 10.1177/0300985818809142

[CR4] Becker LC, Boyer I, Bergfeld WF, Belsito DV, Hill RA, Klaassen CD, Liebler DC, Marks JG, Shank RC, Slaga TJ, Snyder PW, Andersen FA (2016) Safety Assessment of Alumina and Aluminum Hydroxide as Used in Cosmetics. Int J Toxicol 35(3):16S-33S. 10.1177/1091581816677948 27913785 10.1177/1091581816677948

[CR5] Bergfors E, Hermansson G, Nyström Kronander U, Falk L, Valter L, Trollfors B (2014) How common are long-lasting, intensely itching vaccination granulomas and contact allergy to aluminium induced by currently used pediatric vaccines? a prospective cohort study. Eur J Pediatr 173(10):1297–1307. 10.1007/s00431-014-2318-2 24752308 10.1007/s00431-014-2318-2

[CR6] Bougle D, Bureau F, Voirin J, Neuville D, Duhamel J (1992) A cross-sectional study of plasma and urinary aluminum levels in term and preterm infants. J Parenter Enter Nutr 16(2):157–159. 10.1177/0148607192016002157 10.1177/0148607192016002157 1556812

[CR7] Bozynski M, Sedman AB, Naglie RA, Wright EJ (1989) Serial plasma and urinary aluminum levels and tissue loading in preterm twins. J Parenter Enter Nutr 13(4):428–431. 10.1177/0148607189013004428 10.1177/0148607189013004428 2506381

[CR8] Brown RP, Delp MD, Lindstedt SL, Rhomberg LR, Beliles RP (1997) Physiological Parameter Values for Physiologically Based Pharmacokinetic Models. Toxicol Ind Health 13(4):407–484. 10.1177/074823379701300401 9249929 10.1177/074823379701300401

[CR9] Courtney-Martin G, Kosar C, Campbell A, Avitzur Y, Wales PW, Steinberg K, Harrison D, Chambers K (2014) Plasma aluminum concentrations in pediatric patients receiving long-term parenteral nutrition. J Parenter Enter Nutr 39(5):578–585. 10.1177/0148607114531046 10.1177/0148607114531046 24743391

[CR10] Davies B, Morris T (1993) Physiological Parameters in Laboratory Animals and Humans. Pharm Res 10(7):1093–1095. 10.1023/a:1018943613122 8378254 10.1023/a:1018943613122

[CR11] de Ligt R, Westerhout J, Grossouw D, Buters TP, Rissmann R, Burggraaf J, Windhorst AD, Tozer S, Pappa G, Wall B, Bury D, Mason DR, Vaes W (2022) Assessment of dermal absorption of aluminium from a representative antiperspirant formulation using a (26al)al microtracer approach: a follow-up study in humans. Toxicology Research 11(3):511–519. 10.1093/toxres/tfac029 35782644 10.1093/toxres/tfac029 PMC9244721

[CR12] Dirks NL, Meibohm B (2010) Population pharmacokinetics of therapeutic monoclonal antibodies. Clin Pharmacokinet 49(10):633–659. 10.2165/11535960-000000000-00000 20818831 10.2165/11535960-000000000-00000

[CR13] Dixit R, Riviere J, Krishnan K, Andersen M (2003) Toxicokinetics and physiologically based toxicokinetics in toxicology and risk assessment. Journal of Toxicology and Environmental Health, Part B 6(1):1–40. 10.1080/10937400306479 10.1080/10937400306479 12587252

[CR14] EFSA (2008) Safety of aluminium from dietary intake - Scientific Opinion of the Panel on Food Additives, Flavourings, Processing Aids and Food Contact Materials (AFC). EFSA Journal 6(7), 10.2903/j.efsa.2008.754 10.2903/j.efsa.2008.754 PMC10193631 37213837

[CR15] Exley C, Clarkson E (2020) Aluminium in human brain tissue from donors without neurodegenerative disease: A comparison with alzheimer’s disease, multiple sclerosis and autism. Scientific Reports 10(1), 10.1038/s41598-020-64734-6 10.1038/s41598-020-64734-6 PMC7211005 32385326

[CR16] Flarend RE, Hem SL, White JL, Elmore D, Suckow MA, Rudy AC, Dandashli EA (1997) In vivo absorption of aluminium-containing vaccine adjuvants using 26al. Vaccine 15(12–13):1314–1318. 10.1016/s0264-410x(97)00041-8 9302736 10.1016/s0264-410x(97)00041-8

[CR17] García-Patos V, Pujol RM, Alomar A, Cisteró A, Curell R, Fernández-Figueras MT, de Moragas JM (1995) Persistent Subcutaneous Nodules in Patients Hyposensitized With Aluminum-Containing Allergen Extracts. Arch Dermatol 131(12):1421. 10.1001/archderm.1995.01690240085014 7492132

[CR18] Giusti F, Seubert A, Cantisani R, Tortoli M, D’Oro U, Ferlenghi I, Dallai R, Piccioli D (2015) Ultrastructural Visualization of Vaccine Adjuvant Uptake In Vitro and In Vivo. Microsc Microanal 21(4):791–795. 10.1017/s1431927615013744 26223548 10.1017/S1431927615013744

[CR19] Granjon D, Bonny O, Edwards A (2016) A model of calcium homeostasis in the rat. American Journal of Physiology-Renal Physiology 311(5):F1047–F1062. 10.1152/ajprenal.00230.2016 27358053 10.1152/ajprenal.00230.2016

[CR20] Hartung N, Abrams SA, Huisinga W, Weisser K (2024) Consolidated Calcium kinetic rates in a Caucasian population as a function of age and sex. Bone. 10.1016/j.bone.2024.117254 39260784 10.1016/j.bone.2024.117254

[CR21] Hawkins NM, Coffey S, Lawson MS (1994) Potential aluminium toxicity in infants fed special infant formula. J Pediatr Gastroenterol Nutr 19(4):377–381. 10.1097/00005176-199411000-00002 7876989 10.1097/00005176-199411000-00002

[CR22] Hellström HO, Mjöberg B, Mallmin H, Michaëlsson K (2006) No association between the aluminium content of trabecular bone and bone density, mass or size of the proximal femur in elderly men and women. BMC Musculoskeletal Disorders 7(1), 10.1186/1471-2474-7-69 10.1186/1471-2474-7-69 PMC1560132 16928265

[CR23] Hellström HO, Mjöberg B, Mallmin H, Michaëlsson K (2005) The aluminum content of bone increases with age, but is not higher in hip fracture cases with and without dementia compared to controls. Osteoporos Int 16(12):1982–1988. 10.1007/s00198-005-1981-6 16047227 10.1007/s00198-005-1981-6

[CR24] Hethey C, Hartung N, Wangorsch G, Weisser K, Huisinga W (2021) Physiology-based toxicokinetic modelling of aluminium in rat and man. Arch Toxicol 95(9):2977–3000. 10.1007/s00204-021-03107-y 34390355 10.1007/s00204-021-03107-y PMC8380244

[CR25] Hiller J, Göen T, Drexler H, Berking C, Wagner N (2024) Elevated aluminum excretion in patients by long-term subcutaneous immunotherapy - a cross-sectional case-control study. Int J Hyg Environ Health 258:114337. 10.1016/j.ijheh.2024.114337 38461738 10.1016/j.ijheh.2024.114337

[CR26] Hongve D, Johansen S, Andruchow E, Bjertness E, Becher G, Alexander J (1996) Determination of aluminium in samples from bone and liver of elderly norwegians. J Trace Elem Med Biol 10(1):6–11. 10.1016/s0946-672x(96)80002-2 8793817 10.1016/S0946-672X(96)80002-2

[CR27] House E, Esiri M, Forster G, Ince PG, Exley C (2012) Aluminium, iron and copper in human brain tissues donated to the medical research council’s cognitive function and ageing study. Metallomics 4(1):56–65. 10.1039/c1mt00139f 22045115 10.1039/c1mt00139f

[CR28] Huisinga W, Solms A, Fronton L, Pilari S (2012) Modeling Interindividual Variability in Physiologically Based Pharmacokinetics and Its Link to Mechanistic Covariate Modeling. CPT: Pharmacometrics & Systems Pharmacology 1(9):1–10, 10.1038/psp.2012.3 10.1038/psp.2012.3 PMC3603474 23835884

[CR29] ICRP (2002) Basic anatomical and physiological data for use in radiological protection: reference values: Icrp publication 89: Approved by the commission in september 2001. Ann ICRP 32(3–4):1–277. 10.1016/s0146-6453(03)00002-2 14506981

[CR30] Ittel TH, Steinhausen C, Kislinger G, Kinzel S, Nolte E, Sieberth HG (1997) Ultrasensitive analysis of the intestinal absorption and compartmentalization of aluminium in uraemic rats: a 26al tracer study employing accelerator mass spectrometry. Nephrol Dial Transplant 12(7):1369–1375. 10.1093/ndt/12.7.1369 9249771 10.1093/ndt/12.7.1369

[CR31] Janmahasatian S, Duffull SB, Ash S, Ward LC, Byrne NM, Green B (2005) Quantification of lean bodyweight. Clin Pharmacokinet 44(10):1051–1065. 10.2165/00003088-200544100-00004 16176118 10.2165/00003088-200544100-00004

[CR32] Janvier (2024) WISTAR rat growth curve. online resource, https://janvier-labs.com/en/fiche_produit/wistar_rat/

[CR33] Johner SA, Boeing H, Thamm M, Remer T (2015) Urinary 24-h creatinine excretion in adults and its use as a simple tool for the estimation of daily urinary analyte excretion from analyte/creatinine ratios in populations. Eur J Clin Nutr 69(12):1336–1343. 10.1038/ejcn.2015.121 26220572 10.1038/ejcn.2015.121

[CR34] Jones HM, Rowland-Yeo K (2013) Basic Concepts in Physiologically Based Pharmacokinetic Modeling in Drug Discovery and Development. CPT: Pharmacometrics & Systems Pharmacology 2(8):1–12, 10.1038/psp.2013.41 10.1038/psp.2013.41 PMC3828005 23945604

[CR35] Klein G, Alfrey A, Miller N, Sherrard D, Hazlet T, Ament M, Coburn JW (1982) Aluminum loading during total parenteral nutrition. Am J Clin Nutr 35(6):1425–1429. 10.1093/ajcn/35.6.1425 6805302 10.1093/ajcn/35.6.1425

[CR36] Klein GL, Berquist WE, Ament ME, Coburn JW, Miller NL, Alfrey AC (1984) Hepatic aluminum accumulation in children on total parenteral nutrition. J Pediatr Gastroenterol Nutr 3(5):740–743. 10.1097/00005176-198411000-00018 6438295 10.1097/00005176-198411000-00018

[CR37] Krewski D, Yokel RA, Nieboer E, Borchelt D, Cohen J, Harry J, Kacew S, Lindsay J, Mahfouz AM, Rondeau V (2007) Human health risk assessment for aluminium, aluminium oxide, and aluminium hydroxide. Journal of Toxicology and Environmental Health, Part B 10(sup1):1–269. 10.1080/10937400701597766 10.1080/10937400701597766 PMC2782734 18085482

[CR38] Kruger PC, Parsons PJ, Galusha AL, Morrissette M, Recker RR, Howard LJ (2014) Excessive Aluminum Accumulation in the Bones of Patients on Long-Term Parenteral Nutrition: Postmortem Analysis by Electrothermal Atomic Absorption Spectrometry. J Parenter Enter Nutr 38(6):728–735. 10.1177/0148607113491981 10.1177/0148607113491981 23765064

[CR39] Lavielle M (2021) mlxR: Simulation of Longitudinal Data. https://CRAN.R-project.org/package=mlxR, r package version 4.2.0

[CR40] Leggett RW (1992) A Generic Age-Specific Biokinetic Model for Calcium-like Elements. Radiat Prot Dosimetry 41(2–4):183–198. 10.1093/oxfordjournals.rpd.a081254

[CR41] Lin WT, Chen RC, Lu WW, Liu SH, Yang FY (2015) Protective effects of low-intensity pulsed ultrasound on aluminum-induced cerebral damage in alzheimer’s disease rat model. Scientific Reports 5(1), 10.1038/srep09671 10.1038/srep09671 PMC4397698 25873429

[CR42] Lindblad EB (2004) Aluminium adjuvants-in retrospect and prospect. Vaccine 22(27–28):3658–3668. 10.1016/j.vaccine.2004.03.032 15315845 10.1016/j.vaccine.2004.03.032

[CR43] Litov RE, Sickles VS, Chan GM, Springer MA, Cordano A (1989) Plasma Aluminum Measurements in Term Infants Fed Human Milk or a Soy-Based Infant Formula. Pediatrics 84(6):1105–1107. 10.1542/peds.84.6.1105 2587141

[CR44] McDougall SA, Heath MD, Kramer MF, Skinner MA (2016) Analysis of aluminium in rat following administration of allergen immunotherapy using either aluminium or microcrystalline-tyrosine-based adjuvants. Bioanalysis 8(6):547–556. 10.4155/bio.16.10 26915397 10.4155/bio.16.10

[CR45] McLachlan DRC, Alexandrov PN, Walsh WJ, Pogue AI, Percy ME, Kruck TPA, Fang Z, Scharfman N, Jaber V, Zhao Y, Li W, Lukiw WJ (2018) Aluminum in Neurological Disease - a 36 Year Multicenter Study. Journal of Alzheimer’s Disease & Parkinsonism 08(06), 10.4172/2161-0460.1000457 10.4172/2161-0460.1000457 PMC6550484 31179161

[CR46] Moreno A, Domínguez C, Ballabriga A (1994) Aluminium in the neonate related to parenteral nutrition. Acta Paediatr 83(1):25–29. 10.1111/j.1651-2227.1994.tb12947.x 8193468 10.1111/j.1651-2227.1994.tb12947.x

[CR47] Newton D, Talbot RJ (2012) Long-term retention of injected aluminium-26. Human & Experimental Toxicology 31(12):1195–1198. 10.1177/0960327112441038 22549096 10.1177/0960327112441038

[CR48] O’Flaherty EJ (1991a) Physiologically based models for bone-seeking elements: I. Rat skeletal and bone growth. Toxicology and Applied Pharmacology 111(2):299–312, 10.1016/0041-008x(91)90032-a 10.1016/0041-008x(91)90032-a 1957314

[CR49] O’Flaherty EJ (1991b) Physiologically based models for bone-seeking elements: II. Kinetics of lead disposition in rats. Toxicology and Applied Pharmacology 111(2):313–331, 10.1016/0041-008x(91)90033-b 10.1016/0041-008x(91)90033-b 1957315

[CR50] O’Flaherty EJ (1991c) Physiologically based models for bone-seeking elements: III. Human skeletal and bone growth. Toxicology and Applied Pharmacology 111(2):332–341, 10.1016/0041-008x(91)90034-c 10.1016/0041-008x(91)90034-c 1957316

[CR51] O’Flaherty EJ (1993) Physiologically based models for bone-seeking elements: IV. Kinetics of Lead Disposition in Humans. Toxicology and Applied Pharmacology 118(1):16–29, 10.1006/taap.1993.1004 10.1006/taap.1993.1004 8430422

[CR52] Pertinez H, Chenel M, Aarons L (2013) A physiologically based pharmacokinetic model for strontium exposure in rat. Pharm Res 30(6):1536–1552. 10.1007/s11095-013-0991-x 23543304 10.1007/s11095-013-0991-x

[CR53] Poulin P, Theil F (2002) Prediction of Pharmacokinetics Prior to In Vivo Studies. 1. Mechanism-Based Prediction of Volume of Distribution. Journal of Pharmaceutical Sciences 91(1):129–156, 10.1002/jps.10005 10.1002/jps.10005 11782904

[CR54] Priest ND (2004) The biological behaviour and bioavailability of aluminium in man, with special reference to studies employing aluminium-26 as a tracer: review and study update. J Environ Monit 6(5):375–403. 10.1039/b314329p 15152306 10.1039/b314329p

[CR55] R Core Team (2022) R: A Language and Environment for Statistical Computing. R Foundation for Statistical Computing, https://www.R-project.org/

[CR56] Rahil-Khazen R, Bolann BJ, Myking A, Ulvik RJ (2002) Multi-element analysis of trace element levels in human autopsy tissues by using inductively coupled atomic emission spectrometry technique (icp-aes). J Trace Elem Med Biol 16(1):15–25. 10.1016/s0946-672x(02)80004-9 11878748 10.1016/S0946-672X(02)80004-9

[CR57] Rhodin MM, Anderson BJ, Peters AM, Coulthard MG, Wilkins B, Cole M, Chatelut E, Grubb A, Veal GJ, Keir MJ, Holford N (2009) Human renal function maturation: a quantitative description using weight and postmenstrual age. Pediatr Nephrol 24(1):67–76. 10.1007/s00467-008-0997-5 18846389 10.1007/s00467-008-0997-5

[CR58] Richardson RB (2010) A physiological skeletal model for radionuclide and stable element biokinetics in children and adults. Health Phys 99(4):471–482. 10.1097/hp.0b013e3181d0cd4a 20838088 10.1097/HP.0b013e3181d0cd4a

[CR59] Rohatgi A (2022) WebPlotDigitizer [Online]. https://automeris.io/WebPlotDigitizer

[CR60] Roider G, Drasch G (1999) Concentration of aluminum in human tissues - investigations on an occupationally non-exposed population in Southern Bavaria (Germany). Trace Elem Electrolytes 16(2):77–86

[CR61] Sedman AB, Klein GL, Merritt RJ, Miller NL, Weber KO, Gill WL, Anand H, Alfrey AC (1985) Evidence of aluminum loading in infants receiving intravenous therapy. N Engl J Med 312(21):1337–1343. 10.1056/nejm198505233122101 3921839 10.1056/NEJM198505233122101

[CR62] Shirley D, Lote C (2005) Renal handling of aluminium. Nephron. Physiology 101(4):99-p103. 10.1159/000088331 10.1159/000088331 16174991

[CR63] Sofronescu AG (2019) Serum Calcium: Reference Range. https://emedicine.medscape.com/article/2087447-overview?form=fpf

[CR64] Stoehr G, Luebbers K, Wilhelm M, Hoelzer J, Ohmann C (2006) Aluminum load in icu patients during stress ulcer prophylaxis. Eur J Intern Med 17(8):561–566. 10.1016/j.ejim.2006.07.014 17142175 10.1016/j.ejim.2006.07.014

[CR65] Talbot RJ, Newton D, Priest ND, Austin JG, Day JP (1995) Inter-subject variability in the metabolism of aluminium following intravenous injection as citrate. Human & Experimental Toxicology 14(7):595–599. 10.1177/096032719501400707 7576820 10.1177/096032719501400707

[CR66] Tang S, Parsons PJ, Perl D (1999) Longitudinal and lateral variations in the aluminum concentration of selected caprine, bovine, and human bone samples. Biol Trace Elem Res 68(3):267–279. 10.1007/bf02783908 10328341 10.1007/BF02783908

[CR67] Tietz T, Lenzner A, Kolbaum AE, Zellmer S, Riebeling C, Gürtler R, Jung C, Kappenstein O, Tentschert J, Giulbudagian M, Merkel S, Pirow R, Lindtner O, Tralau T, Schäfer B, Laux P, Greiner M, Lampen A, Luch A, Wittkowski R, Hensel A (2019) Aggregated aluminium exposure: risk assessment for the general population. Arch Toxicol 93(12):3503–3521. 10.1007/s00204-019-02599-z 31659427 10.1007/s00204-019-02599-z

[CR68] Veiga M, Bohrer D, Banderó CR, Oliveira SM, do Nascimento PC, Mattiazzi P, Mello CF, Lenz QF, Oliveira MS (2013) Accumulation, elimination, and effects of parenteral exposure to aluminum in newborn and adult rats. Journal of Inorganic Biochemistry 128:215–220. 10.1016/j.jinorgbio.2013.07.028 10.1016/j.jinorgbio.2013.07.028 23916521

[CR69] Verdier F, Burnett R, Michelet-Habchi C, Moretto P, Fievet-Groyne F, Sauzeat E (2005) Aluminium assay and evaluation of the local reaction at several time points after intramuscular administration of aluminium containing vaccines in the cynomolgus monkey. Vaccine 23(11):1359–1367. 10.1016/j.vaccine.2004.09.012 15661384 10.1016/j.vaccine.2004.09.012

[CR70] Washington N, Wilson C (1986) Antacids: physiology versus pharmaceutics. Int J Pharm 28(2–3):249–260. 10.1016/0378-5173(86)90251-6

[CR71] Weisser K, Stübler S, Matheis W, Huisinga W (2017) Towards toxicokinetic modelling of aluminium exposure from adjuvants in medicinal products. Regul Toxicol Pharmacol 88:310–321. 10.1016/j.yrtph.2017.02.018 28237896 10.1016/j.yrtph.2017.02.018

[CR72] Weisser K, Göen T, Oduro JD, Wangorsch G, Hanschmann K, Keller-Stanislawski B (2019) Aluminium in plasma and tissues after intramuscular injection of adjuvanted human vaccines in rats. Arch Toxicol 93(10):2787–2796. 10.1007/s00204-019-02561-z 31522239 10.1007/s00204-019-02561-z

[CR73] Weisser K, Göen T, Oduro JD, Wangorsch G, Hanschmann KO, Keller-Stanislawski B (2020) Aluminium from adjuvanted subcutaneous allergen immunotherapeutics in rats is mainly detected in bone. Allergy 75(1):215–217. 10.1111/all.13982 31306489 10.1111/all.13982

[CR74] Yokel RA, McNamara PJ (2001) Aluminium Toxicokinetics: An Updated MiniReview. Pharmacology & Toxicology 88(4):159–167. 10.1111/j.1600-0773.2001.880401.x 11322172 10.1034/j.1600-0773.2001.d01-98.x

