# Supplementary material for: Extension and validation of a physiologically based toxicokinetic model for risk assessment of aluminium exposure in humans
Source: Arch Toxicol. 2025 Apr 19;99(6):2379–95. doi: 10.1007/s00204-025-04031-1 (PMC12185619; doi:10.1007/s00204-025-04031-1)
Supplement: Supplementary file 1 — (pdf 1230 KB) [file 204_2025_4031_MOESM1_ESM.pdf]

Supplementary material

S1 Model equations

We modelled the rate of change of the amount of Al in blood (addCit, Mix) and tissues ($\text{tis} \in \{\text{liv}, \text{spl}, \text{mus}, \text{bon}, \text{bra}, \text{kid}, \text{rob}\}$) by the following system of ordinary differential equations:

$$\frac{\mathrm{d}}{\mathrm{d}t} A_{\text{gut}} = -k_{\text{gut2blo}} \cdot A_{\text{gut}} \tag{S1}$$

$$\frac{\mathrm{d}}{\mathrm{d}t} A_{\text{gut,BW}} = \left(k_{\text{gut2blo}} + \frac{\mathrm{d}}{\mathrm{d}t} \log \text{BW} \right) \cdot A_{\text{gut,BW}} \tag{S2}$$

$$\begin{aligned} \frac{\mathrm{d}}{\mathrm{d}t} A_{\text{addCit}} &= -k_{\text{Cit2Mix}} \cdot A_{\text{addCit}} - \frac{\text{CL}_{\text{Cit}}}{V_{\text{blo}}} \cdot A_{\text{addCit}} \\ &\quad + \frac{A_{\text{addCit}}}{A_{\text{blo}}} \sum_{\text{tis}} k_{\text{tis2blo}} \cdot A_{\text{tis}} - \sum_{\text{tis}} k_{\text{blo2tis}} \cdot A_{\text{addCit}} \end{aligned} \tag{S3}$$

$$\begin{aligned} \frac{\mathrm{d}}{\mathrm{d}t} A_{\text{Mix}} &= k_{\text{Cit2Mix}} \cdot A_{\text{addCit}} - \frac{\text{CL}_{\text{Mix}}}{V_{\text{blo}}} \cdot A_{\text{Mix}} \\ &\quad + F \cdot k_{\text{gut2blo}} \cdot \big(A_{\text{gut}} + A_{\text{gut,BW}} \cdot \text{BW} \big) \\ &\quad + \frac{A_{\text{Mix}}}{A_{\text{blo}}} \sum_{\text{tis}} k_{\text{tis2blo}} \cdot A_{\text{tis}} - \sum_{\text{tis}} k_{\text{blo2tis}} \cdot A_{\text{Mix}} \end{aligned} \tag{S4}$$

$$\frac{\mathrm{d}}{\mathrm{d}t} A_{\text{tis}} = k_{\text{blo2tis}} \cdot A_{\text{blo}} - k_{\text{tis2blo}} \cdot A_{\text{tis}} \tag{S5}$$

$$\frac{\mathrm{d}}{\mathrm{d}t} A_{\text{uri}} = \frac{1}{V_{\text{blo}}} \left(\text{CL}_{\text{Cit}} \cdot A_{\text{addCit}} + \text{CL}_{\text{Mix}} \cdot A_{\text{Mix}} \right) \tag{S6}$$

with bioavailability F and clearance CL and $A_{\text{blo}} = A_{\text{addCit}} + A_{\text{Mix}}$.

Two gut compartments were considered to allow for both absolute as well as relative per body weight (BW) oral dosing, used to represent drugs and food, respectively. The second gut compartment was necessary because Al intake via food was modelled as a continuous intake relative to a (changing) BW, which cannot be converted to an absolute intake rate independently of the BW evolution. For relative dosing, the impact of changing body weight during growth resulted in a second term in Eq. S2.

S2 Details on parametrisation of the bone module in rats

Starting point are the allometric relationships (BW in g) reported in O'Flaherty (1991a,b),

$$
\begin{aligned}
W_{\mathrm{Ca}} &= 0.002\,\mathrm{g} \cdot \mathrm{BW}^{1.3}, \\
W_{\mathrm{bon}} &= 0.0801\,\mathrm{g} \cdot \mathrm{BW}^{0.983}, \\
W_{\mathrm{mar}} &= 0.0469\,\mathrm{g} \cdot \mathrm{BW}^{0.866}, \\
W_{\mathrm{mfb}} &= 0.0373\,\mathrm{g} \cdot \mathrm{BW}^{1.06}, \\
V_{\mathrm{mfb}} &= 0.0252\,\mathrm{mL} \cdot \mathrm{BW}^{1.02},
\end{aligned}
$$

with V and W denoting volumes and weights, respectively, 'mar' being bone marrow, 'mfb' marrow-free bone, and 'bon' the model bone compartment (cartilage-free bone). With marrow density $D_{\mathrm{mar}} = 1$ kg/L and volume $V_{\mathrm{mar}} = W_{\mathrm{mar}}/D_{\mathrm{mar}}$, the intermediate quantities

$$V_{\mathrm{bon}} = V_{\mathrm{mfb}} + V_{\mathrm{mar}}, \tag{S7}$$

$$D_{\mathrm{bon}} = \frac{W_{\mathrm{bon}}}{V_{\mathrm{bon}}} \tag{S8}$$

were derived. We then considered the rat growth model from O'Flaherty (1991a),

$$\mathrm{BW(age)} = 5\,\mathrm{g} + 375\,\mathrm{g} \cdot \frac{\exp(\mathrm{age}) - 1}{\exp(1.6\,\mathrm{mo}) + \exp(\mathrm{age}) - 1} + 8.8\,\frac{\mathrm{g}}{\mathrm{mo}} \cdot \mathrm{age}, \tag{S9}$$

with age specified in weeks. For each of the two rat physiologies we considered (obtained from Brown et al. 1997), age was chosen such that BW(age) matches the reported body weights (250 g and 475 g, respectively). Then, we used the expressions for fractional bone formation rate (fbfr) and fractional bone resorption rate (fbrr) from O'Flaherty (1991a),

$$
\begin{aligned}
\mathrm{fbfr} &= \frac{0.003}{\mathrm{day}} + \frac{0.321}{\mathrm{day}} \cdot \exp\left(-\frac{0.2}{\mathrm{day}} \cdot \mathrm{age}\right) + \frac{0.081}{\mathrm{day}} \cdot \exp\left(-\frac{0.021}{\mathrm{day}} \cdot \mathrm{age}\right), \\
\mathrm{fbrr} &= \mathrm{fbfr} - \frac{\mathrm{d}}{\mathrm{d\ age}}\Big(V_{\mathrm{mfb}}(\mathrm{BW(age)})\Big),
\end{aligned}
$$

with age in days and using the above growth model, Eq. S9. This finally leads to the relationships

$$v_+^{\mathrm{Ca}} = \mathrm{fbfr} \cdot W_{\mathrm{Ca}}; \qquad v_-^{\mathrm{Ca}} = \mathrm{fbrr} \cdot W_{\mathrm{Ca}}.$$

The physiological parameters D_{bon}, V_{bon}, obtained from Brown et al. (1997) in the previous model by Hethey et al. (2021), were replaced by the corresponding values from Eqs. S7–S8 (as a consequence, V_{rob} was updated as well).

S3 Human bone composition

According to ICRP (2002), total skeleton ("tot") is composed of the sum of bone tissue ("tis"), cartilage ("car"), bone marrow, teeth, and miscellaneous (taken together as "res").

Since O'Flaherty (1991c) reported that cartilage is not quantitatively important as a determinant of Ca-mimicking elements, we defined the bone compartment ("bon") in the Al PBTK model to be comprised of cartilage-free total skeleton ("cfb"). Bone density (mass of bone per unit volume) is a parameter necessary for transformation of model predictions for Al bone content in µg/L into µg/g. Therefore, mass, volume and density of cartilage-free total skeleton were needed for all age groups.

The age-dependent fraction of bone tissue mass (sum of cortical and trabecular bone) in cartilage-free total skeleton decreases from 70.8% in newborns to 58.5% in male adults, whereas density of mineralised bone tissue increases from 1.65 to 1.9 g/L (see Table S1 and ICRP 2002). As densities of cortical and trabecular mineralised bone tissue are described as "only slightly different" (ICRP 2002), the density of hydrated bone tissue (cortical bone) was used for bone tissue D_{tis}.

Furthermore, density of the whole fresh skeleton in a reference adult is given as D_{tot}=1.3 g/mL and that of fresh cartilage as D_{car}=1.1 g/mL (ICRP 2002). Using these values along with the reported weights of skeletal components, density of cartilage-free bone in adults can be calculated as

$$D_{\text{cfb}} = \frac{W_{\text{cfb}}}{V_{\text{cfb}}} = \frac{W_{\text{tot}} - W_{\text{car}}}{V_{\text{tot}} - V_{\text{car}}} = \frac{W_{\text{tot}} - W_{\text{car}}}{\frac{W_{\text{tot}}}{D_{\text{tot}}} - \frac{W_{\text{car}}}{D_{\text{car}}}}.$$

However, for infants and children, no reference value for D_{tot} is given. To calculate D_{cfb} in this age range, we first calculated the density of rest of bone in adults,

$$D_{\text{res}} = \frac{W_{\text{res}}}{V_{\text{res}}} = \frac{W_{\text{cfb}} - W_{\text{tis}}}{V_{\text{cfb}} - V_{\text{tis}}} = \frac{W_{\text{cfb}} - W_{\text{tis}}}{V_{\text{cfb}} - \frac{W_{\text{tis}}}{D_{\text{tis}}}},$$

whilch yielded $D_{\text{res}} = 0.933$ g/mL for men and $D_{\text{res}} = 0.944$ g/mL for women.

Using D_{res} (men) for 15 year-old boys and D_{res} (women) for all girls and boys below 10 years of age, we then calculated D_{cfb} for all reference ages via

$$D_{\text{cfb}} = \frac{W_{\text{cfb}}}{V_{\text{cfb}}} = \frac{W_{\text{tis}} + W_{\text{res}}}{V_{\text{tis}} + V_{\text{res}}} = \frac{W_{\text{tis}} + W_{\text{res}}}{\frac{W_{\text{tis}}}{D_{\text{tis}}} + \frac{W_{\text{res}}}{D_{\text{res}}}}$$

see Table S1.

S4 Derivation of dry-to-wet weight conversion factors

For all tissues except bone (brain, liver and kidney, resp.) the published fractional water content (W) of the organs (77, 75, and 78 %, resp.; Poulin and

Table S1 Weights, volumes and densities of mineralised bone tissue and cartilage-free total skeleton for humans of different ages

	Skeletal weight (g)					Volume (mL)			Dens. (g/mL)	
Age	tot*	car*	cfb#	tis*	res#	cfb#	tis#	res#	tis*	cfb#
newborn	370	130	240	170	70	177	103	74	1.65	1.35
1 y	1170	360	810	590	220	589	355	233	1.66	1.38
5 y	2430	600	1830	1260	570	1346	741	605	1.70	1.36
10 y	4500	820	3680	2300	1380	2742	1278	1464	1.75	1.34
15 y (m)	7950	1140	6810	4050	2760	5210	2250	2960	1.80	1.31
15 y (f)	7180	920	6260	3700	2560	4771	2056	2716	1.80	1.31
adult (m)	10500	1100	9400	5500	3900	7077	2895	4182	1.90	1.33
adult (f)	7800	900	6900	4000	2900	5182	2105	3077	1.90	1.33

Abbreviations: Dens., density; tot, total skeleton (tis+car+res); car, cartilage; cfb, cartilage-free total skeleton (tis+res); tis, mineralized bone tissue only; res, rest of bone; m, male; f, female; *, reference values from ICRP (2002); #, derived as described in the text.

Theil 2002) was used to convert the data from dry to wet weight:

$$1\ \frac{\text{g Al}}{\text{g dry tissue}} \cdot (1-W)\ \frac{\text{g dry tissue}}{\text{g wet tissue}} = (1-W)\ \frac{\text{g Al}}{\text{g wet tissue}}.$$

In order to convert Al bone content in marrow-free human bone samples of adults reported in literature (Hellström et al. 2005; Kruger et al. 2014; Klein et al. 1982) into Al content based on wet model bone, the following equation was used (cfb: cartilage-free bone, mfb: marrow- and cartilage-free bone):

$$1\ \frac{\text{g Al}}{\text{g dry mfb}} \cdot 0.85\ \frac{\text{g dry mfb}}{\text{g dry cfb}} \cdot 0.585\ \frac{\text{g dry cfb}}{\text{g wet cfb}} = 0.497\ \frac{\text{g Al}}{\text{g wet cfb}}$$

The calculation is based on the following assumptions: 1) bone sample preparation resulted in marrow- and cartilage-free bone tissue, i.e. samples for Al measurement consisted of marrow-free (trabecular) bone tissue; 2) Al is solely present in bone tissue and not in marrow or cartilage (O'Flaherty 1991c); 3) fractional water content of bone tissue in an average adult is about 15% (O'Flaherty 1991c, Table 2); 4) weight ratio of wet bone tissue/cartilage-free total skeleton is 58.5% in adults (calculated from ICRP 2002). In contrast to Hethey et al. (2021), where model bone comprised wet total skeleton according to ICRP (2002), in our Al PBTK model the bone compartment represents cartilage-free total skeleton and hence, fractions of bone tissue in cartilage-free total skeleton (and not total skeleton) were accounted for in the above calculations.

As water content and bone composition is different in newborns and small children, for conversion of bone Al literature data from newborns or children below 10y, a slightly adapted calculation was used:

$$1\ \frac{\text{g Al}}{\text{g dry mfb}} \cdot 0.8\ \frac{\text{g dry mfb}}{\text{g dry cfb}} \cdot 0.708\ \frac{\text{g dry cfb}}{\text{g wet cfb}} = 0.566\ \frac{\text{g Al}}{\text{g wet cfb}}$$

since the water fraction (g/g) of marrow-free bone tissue in newborns/children is about 20% (O'Flaherty 1991a, Table 2) and the weight ratio of wet bone

tissue/cartilage-free total skeleton is about 70.8% in neonates (calculated from ICRP 2002).

In this context, we detected that in the data pool built in Hethey et al. (2021), one small subset of rat bone data by Ittel et al. (1997) had falsely been considered as based on ww although it was referenced to dw. Furthermore, Ittel et al. (1997) described in detail that the samples consisted of marrow-free trabecular bone tissue whereas bone in Hethey et al. (2021) comprised total skeletal bone including bone marrow and cartilage. These error were corrected before re-estimating our model. For this, the Al content of these rat samples was converted according to

$$1 \, \frac{\text{g Al}}{\text{g dry mfb}} \cdot 0.86 \, \frac{\text{g dry mfb}}{\text{g dry cfb}} \cdot 0.70 \, \frac{\text{g dry cfb}}{\text{g wet cfb}} = 0.610 \, \frac{\text{g Al}}{\text{g wet cfb}}$$

based on a water content of bone tissue in rats (230 g) of about 14% and a weight ratio of wet bone tissue/total skeleton of about 70% (O'Flaherty 1991a).

S5 Al intake via food

Realistic values for an average long-term Al exposure from food in different age groups in Europe were taken from EFSA (2008), which report a dietary exposure from 0.2 to 1.5 mg/kg/w in a 60 kg adult. We therefore considered a mean value of 0.8 mg/kg/w for simulation of an average Al food intake in adults.

For infants aged 0-3, 4-6, 7-9 and 10-12 months, (rounded) estimates of potential dietary exposures from infant formulae and other foods manufactured specially for infants of 0.1, 0.2, 0.4 and 0.8 mg/kg/w, respectively, were taken (EFSA 2008).

In children and young people, the potential estimated exposure at the 97.5th percentile ranged from 0.7 mg/kg/w for children aged 3-15 years in France to 2.3 mg/kg/w for toddlers (1.5-4.5 years) and 1.7 mg/kg/w for those aged 4-18 years in the UK (EFSA 2008). Additionally, Tietz et al. (2019) reported a peak of exposure in the age group of 3-6 year old children from French TDS studies (0.64 (normal consumers) — 1.02 mg/kg/w (high consumers)). Therefore, for our simulations we considered 0.8 mg/kg/w as an appropriate average value from the age of 1 year onwards.

S6 Specific data transformations

S6.1 Initial conditions

Summary statistics for Al concentrations in newborns were reported as mean and standard deviation or standard error of measurement, or as a 95% confidence interval for the mean.

If mean M and standard deviation SD were reported in a reference for some organ, corresponding parameters μ and σ^2 of a lognormal distribution $X \sim \log\mathcal{N}(\mu, \sigma^2)$ were determined by matching its moments, i.e.

$$\sigma^2 = \log\left(\frac{\mathrm{SD}^2}{\mathrm{M}^2} + 1\right) \quad \text{and} \quad \mu = \log(\mathrm{M}) - \frac{\sigma^2}{2}.$$

Otherwise, reported values were converted to mean and standard deviation as follows (with n denoting sample size):

– reported standard errors of measurement SEM were converted to standard deviations via $\mathrm{SD} = \mathrm{SEM} \cdot \sqrt{n}$;
– reported 95% confidence intervals for the mean $[L, U]$ were converted to mean M and standard deviation SD via
$$\mathrm{M} = \frac{L+U}{2}, \quad \mathrm{SD} = \sqrt{n} \cdot \frac{U-L}{2 \cdot t_{0.975,n-1}}$$
with $t_{0.975,n-1}$ denoting the 97.5-percentile of a t distribution with $n-1$ degrees of freedom.

S6.2 Urine data by Hiller et al. (2024)

Hiller et al. (2024) reported urine data as µg Al/g creatinine, summarized as mean ± SD for each of the two groups (SCIT and control). Urinary Al excretion rates are then obtained by multiplying these values with sex-averaged creatinine excretion rates

$$f_{\text{women}} \cdot 1.26\,\frac{\mathrm{g}}{\mathrm{d}} + f_{\text{men}} \cdot 1.74\,\frac{\mathrm{g}}{\mathrm{d}},$$

where f_{women} and f_{men} denote the group-specific proportions of the respective sexes and assuming creatinine excretion rates of 1.26 g/d for women and 1.74 g/d for men (Johner et al. 2015).

S6.3 Rat data by Weisser et al. (2019,2020)

Weisser et al. (2019, 2020) measured Al concentrations in plasma, bone and brain in different treatment and control groups. To describe the increase in exposure in treated vs. control animals in the absence of a baseline exposure model (food and initial conditions), excess Al concentrations were calculated as the difference in Al concentrations (mean±standard deviation) in a treated group minus the mean Al concentration in the control group. Consequently, the excess Al concentrations could become negative, either mean−standard deviation of the excess Al concentration or even the mean excess Al concentration.

S7 Supplementary figures and tables

Table S2 Parameter estimates and fixed parameter values in the Al PBTK model. Parameter are assumed to be either lognormally or logit-normally distributed (possibly degenerate, i.e. with variance zero). Reported (typical) values in the second column refer to the population estimate of the fixed effects on the original (non-transformed) scale, including relative standard error (R.S.E.) of the estimated parameter values. Where appropriate, inter-individual variability was quantified as standard deviation of the random effects on the transformed (normal) scale and was denoted by ω.

Parameter	Value	R.S.E. in %	ω	R.S.E. in %
Oral absorption rate constant, log($\mathcal{N}$), in 1/h				
k_{gut2blo}	2.82	96.6	2.47	29
Oral bioavailability, logit($\mathcal{N}$), dimensionless				
F	0.00168	22	0.984	15
Uptake coefficients, logit($\mathcal{N}$), dimensionless				
I_{lsk}	0.00109	39	1.36	22
I_{mus}	0.0113	23	0	fixed
I_{bra}	$1.85 \cdot 10^{-5}$	11	0	fixed
I_{rob}	1	fixed	0	fixed
Retention coefficients, log($\mathcal{N}$), dimensionless				
K_{lsk}	$7.56 \cdot 10^{4}$	64	0	fixed
K_{mus}	110	17	0	fixed
K_{bra}	inf	fixed	0	fixed
K_{rob}	0.55	4	0	fixed
Bone module parameters, log($\mathcal{N}$), dimensionless				
α	0.566	25	0.883	19
β	1	fixed	0.455	65
Equilibration rate constant, log($\mathcal{N}$), in 1/h				
k_{Cit2Mix}	0.0309	25	0	fixed
Effective ultrafiltrable fractions, log($\mathcal{N}$), dimensionless				
fu_{Cit}	1	fixed	0	fixed
fu_{Mix}	0.1	fixed	0	fixed
Glomerular filtration rate, log($\mathcal{N}$), in L/h				
$\text{GFR}_{\text{human}}$	Eq. 2	fixed	0.436	25
GFR_{rat}	Eq. 3	fixed	0.864	24
Blood to plasma concentration ratio, dimensionless				
BP	$(1 - \text{Hct})$	fixed	0	fixed

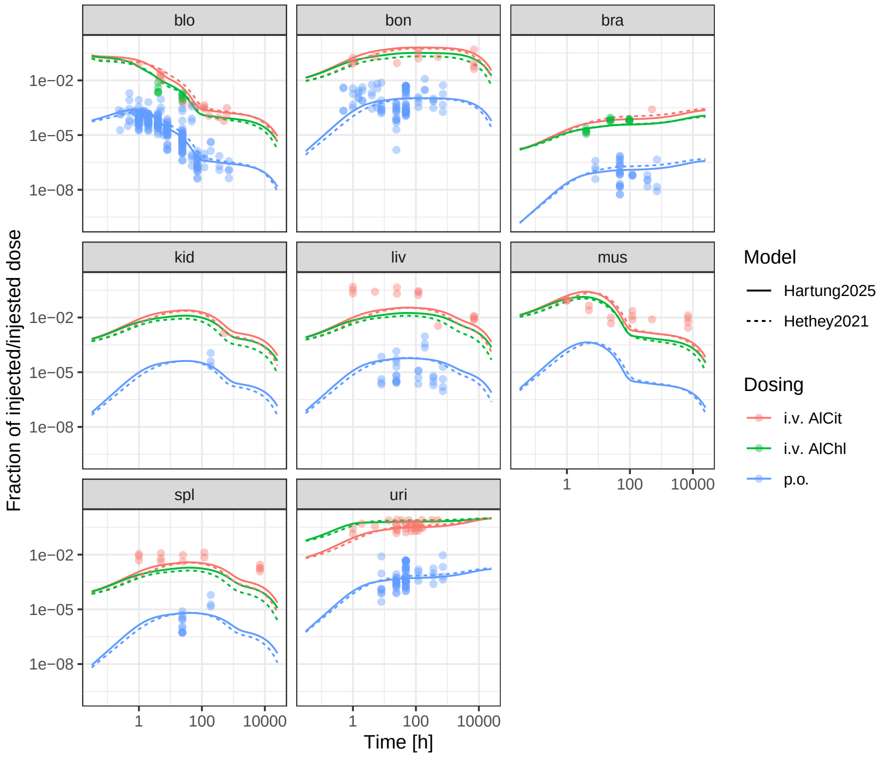

Fig. S1 Impact of model update on fit to ^{26}Al rat data. Abbreviations: blo, blood or plasma; bon, bone; bra, brain; kid, kidney; liv, liver; mus, muscle; spl, spleen; uri, urine.

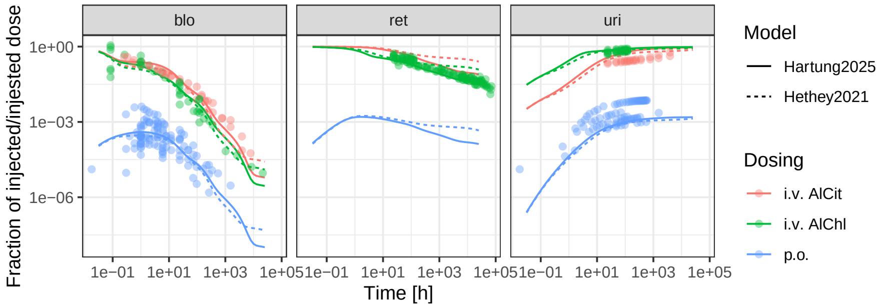

Fig. S2 Impact of model update on fit to ^{26}Al human data. Abbreviations: blo, blood or plasma; ret, whole-body retention; uri, urine.

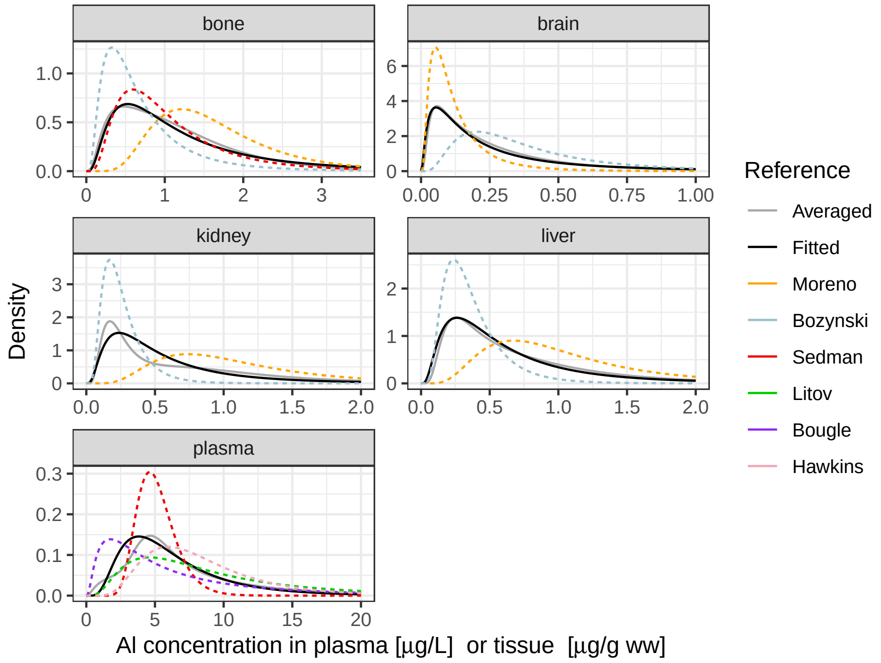

Fig. S3 Distributions of reference Al concentrations in healthy human newborns. To derive the fitted distribution, values reported in different literature sources were matched to a lognormal distribution (colored dashed lines). Subsequently, these lognormal distributions were averaged (grey solid line) and the final fitted lognormal distribution (black solid line) was matched to the averaged distributions. Abbreviations: ww, wet weight.

Table S3 Overview on rat adjuvant data from Weisser et al. (2019, 2020), using their nomenclature for products or preparations.

Product / preparation	Route	Adjuvant type	Al dose
P2	s.c.	AH in situ	1000 μg
P1	s.c.	AH (Alhydrogel)	1130 μg
pAH	s.c.	AH (Alhydrogel)	1250 μg
pAP	i.m.	AP (Adju-Phos)	1250 μg
pAH	i.m.	AH (Alhydrogel)	1250 μg
V1	i.m.	AH (Alhydrogel)	600 μg
V2	i.m.	AH (Alhydrogel) + AP (Adju-Phos)	500 μg+320 μg
V3	i.m.	AP (Adju-Phos)	500 μg

Abbreviations: s.c., subcutaneous; i.m., intramuscular; (p)AH, (plain) aluminium hydroxide; (p)AP, (plain) aluminium phosphate; P1/2, allergen products 1/2; V1-3, vaccine products 1-3.

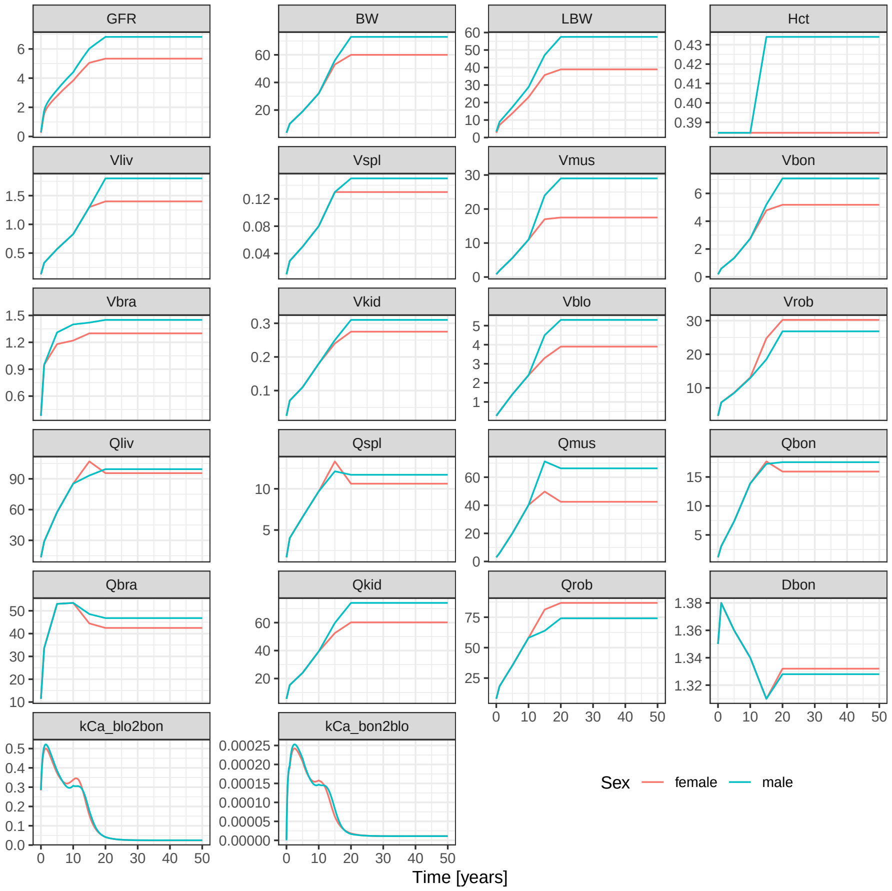

Fig. S4 Age-related changes in physiological parameters. Units: [kg] for (lean) body weight `(L)BW`, [L/h] for glomerular filtration rate `GFR` and blood flows `Q*`, [L] for organ volumes `V*`, [kg/L] for bone density `Dbon`, unitless for hematocrit `Hct`, [1/h] for Ca uptake and release rate constants `kCa_*`.

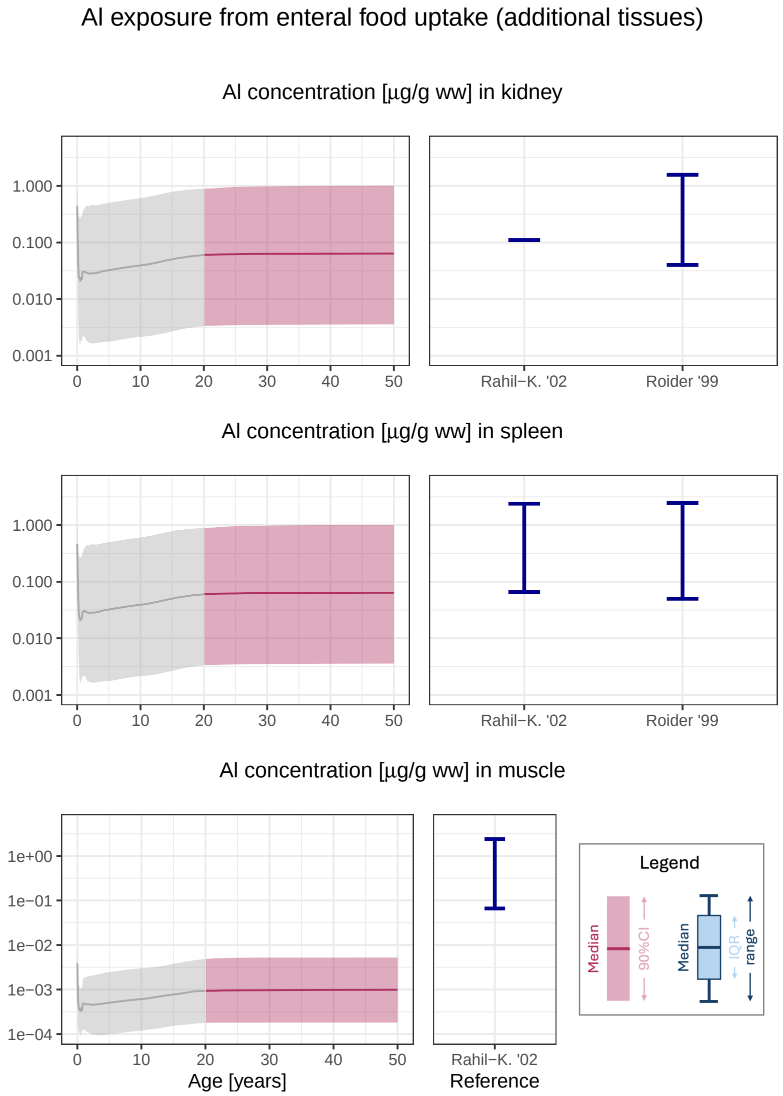

Fig. S5 Reference Al concentrations from enteral uptake via food in kidney, spleen and muscle. (Calculated) median or range are displayed as reported in the respective literature source. All reference values are from adult populations. Abbreviations: ww, wet weight; CI, confidence interval; IQR, interquartile range; Rahil-K., Rahil-Khazen.

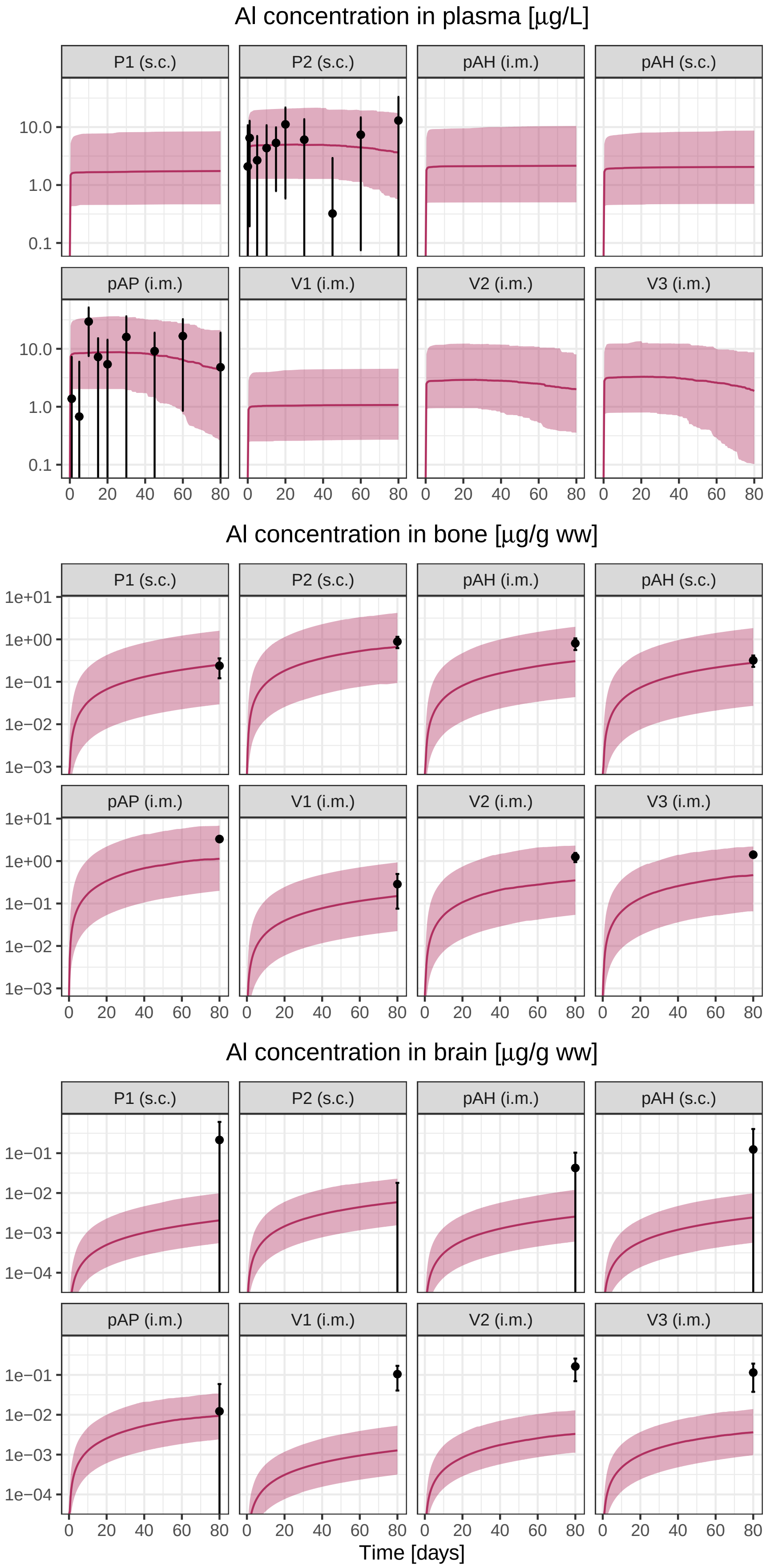

Fig. S6 Simulated and observed Al exposure in rats after administration of different adjuvant products in plasma (top), bone (middle) and brain (bottom). See Table S3 for input kinetics. Simulated Al exposure is via adjuvants only, and data represent excess Al exposure compared to a control group, displayed as mean±SD (note that mean−SD or mean can become negative this way, and whiskers extend to the bottom plot range in this case).

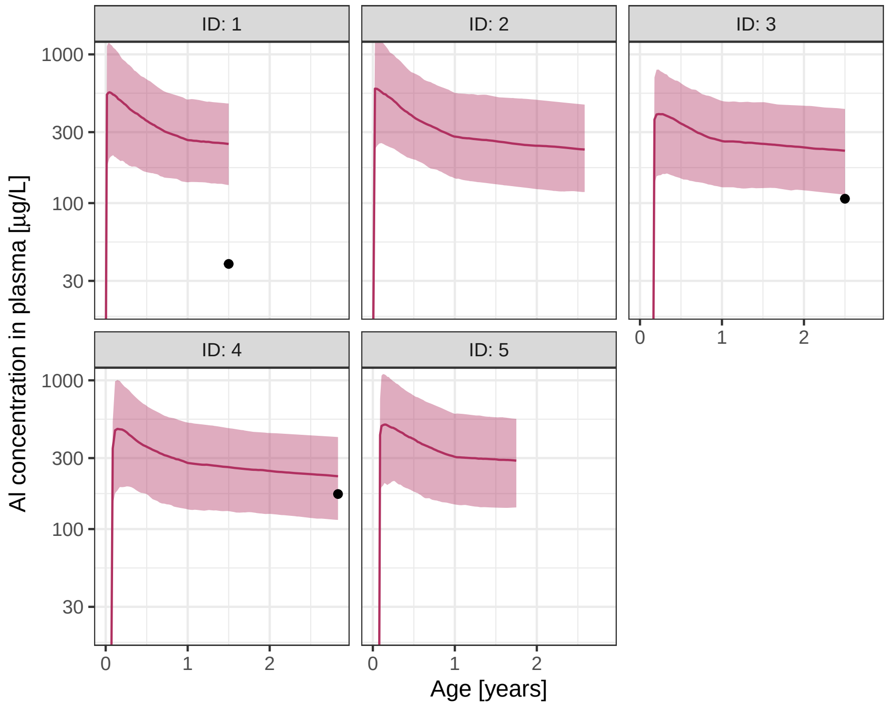

Fig. S7 Plasma simulations vs. observations for the five individuals from Klein et al. (1984), three of which had plasma concentrations measured.

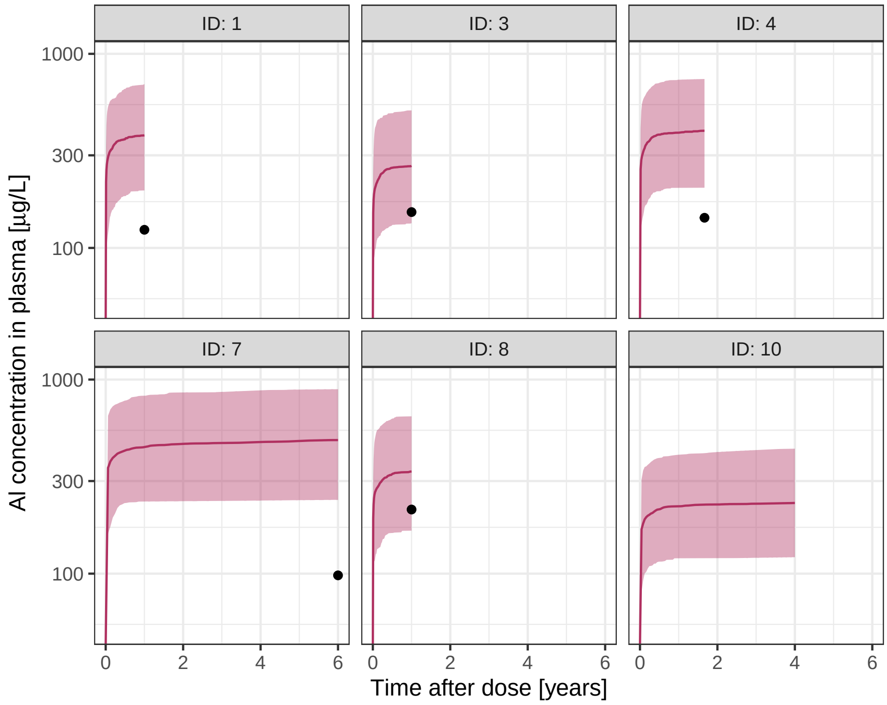

Fig. S8 Plasma simulations vs. observations for the six individuals from Klein et al. (1982), five of which had plasma concentrations measured.

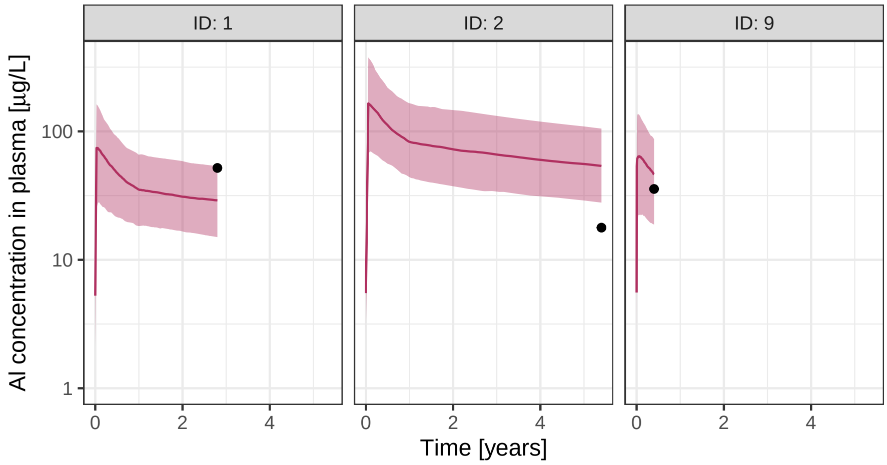

Fig. S9 Plasma simulations vs. observations for three individuals from Courtney-Martin et al. (2014)

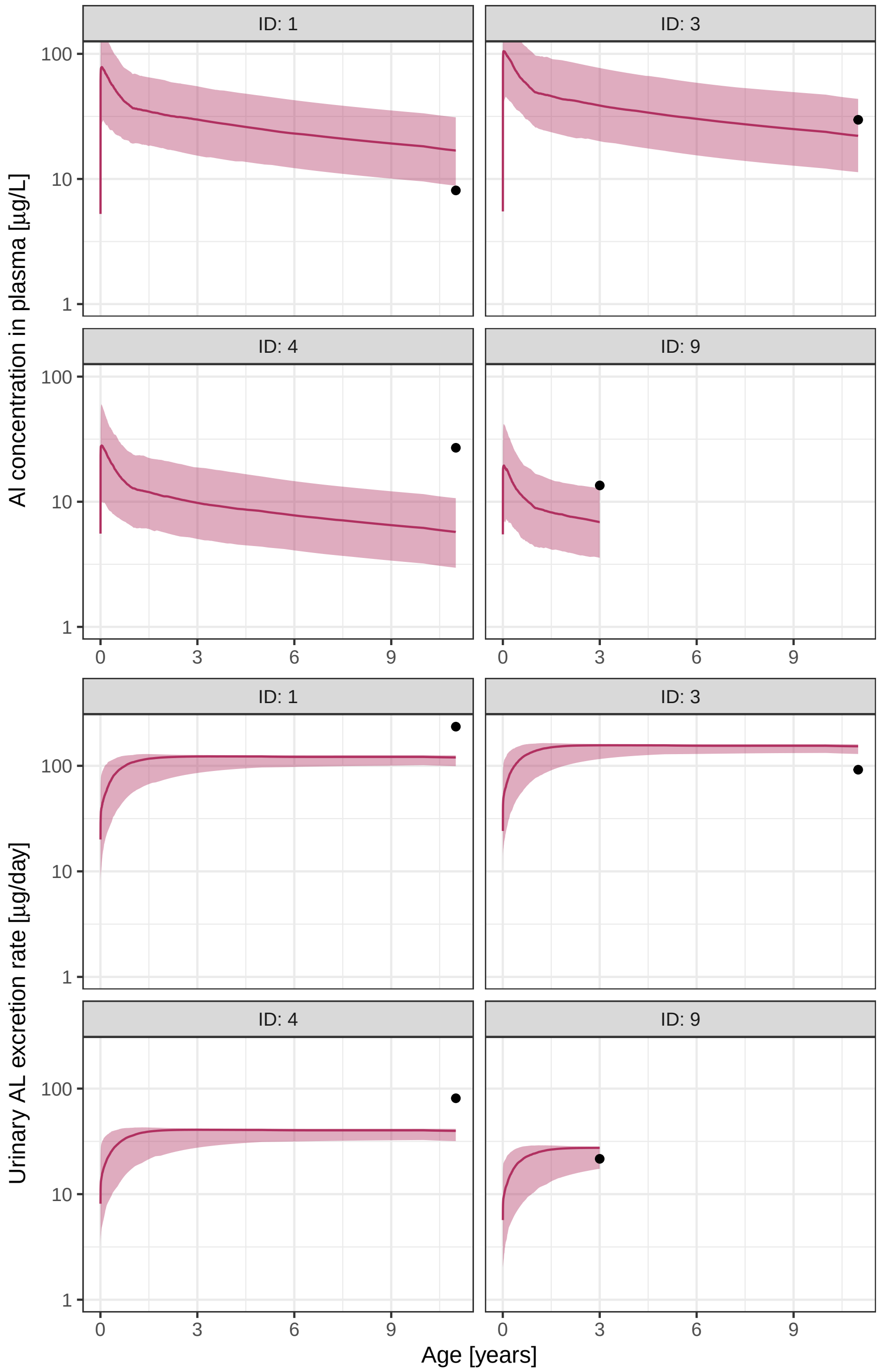

Fig. S10 Model validation vs. data by Advenier et al. (2003) in plasma (top) and urine (bottom).
